# Effect of Oxidation on Vivianite Dissolution Rates and Mechanism

**DOI:** 10.1021/acs.est.4c04809

**Published:** 2024-08-16

**Authors:** Rouven Metz, Naresh Kumar, Walter D. C. Schenkeveld, Martin Obst, Andreas Voegelin, Stefan Mangold, Stephan M. Kraemer

**Affiliations:** † Centre for Microbiology and Environmental Systems Science, Department for Environmental Geosciences, University of Vienna, Josef-Holaubek-Platz 2, 1090 Vienna, Austria; ‡ Soil Chemistry, Wageningen University and Research, Droevendaalsesteeg 3, 6708 PB Wageningen, The Netherlands; § Experimental Biogeochemistry, BayCEER, University of Bayreuth, Dr. Hans-Frisch-Straße 1-3, 95448 Bayreuth, Germany; ∥ Swiss Federal Institute of Aquatic Science and Technology, Department of Water Resources and Drinking Water, Eawag, Ueberlandstrasse 133, CH-8600 Duebendorf, Switzerland; ⊥ Karlsruhe Institute of Technology, Institute for Photon Science and Synchrotron Radiation, Hermann-von-Helmholtz Platz 1, D-76344 Eggenstein-Leopoldshafen, Germany

**Keywords:** mineral transformation, oxidation kinetics, core–shell structure, metastability, amorphous iron phosphate, santabarbaraite

## Abstract

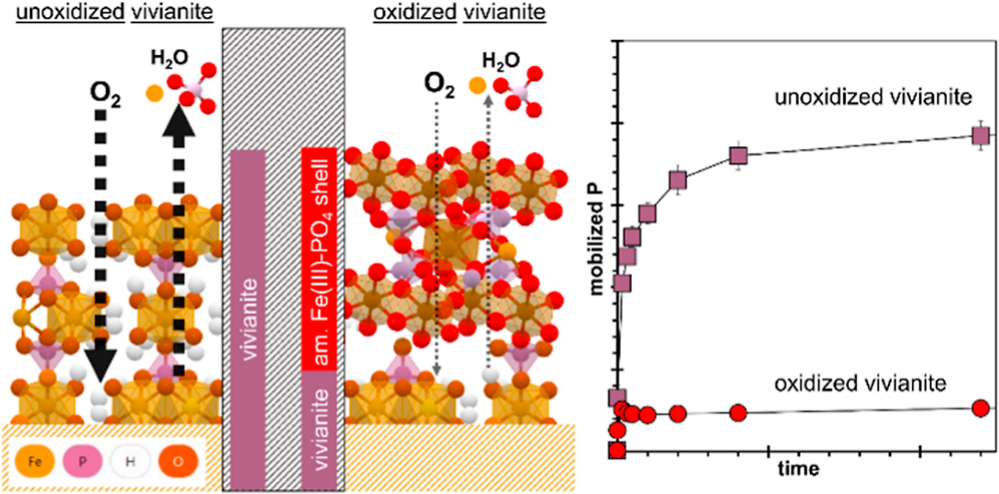

The interest in the mineral vivianite (Fe_3_(PO_4_)_2_·8H_2_O) as a more sustainable P resource has grown significantly in recent years owing to its efficient recovery from wastewater and its potential use as a P fertilizer. Vivianite is metastable in oxic environments and readily oxidizes. As dissolution and oxidation occur concurrently, the impact of oxidation on the dissolution rate and mechanism is not fully understood. In this study, we disentangled the oxidation and dissolution of vivianite to develop a quantitative and mechanistic understanding of dissolution rates and mechanisms under oxic conditions. Controlled batch and flow-through experiments with pristine and preoxidized vivianite were conducted to systematically investigate the effect of oxidation on vivianite dissolution at various pH-values and temperatures. Using X-ray absorption spectroscopy and scanning transmission X-ray microscopy techniques, we demonstrated that oxidation of vivianite generated a core–shell structure with a passivating oxidized amorphous Fe(III)–PO_4_ surface layer and a pristine vivianite core, leading to diffusion-controlled oxidation kinetics. Initial (<1 h) dissolution rates and concomitant P and Fe release (∼48 h) decreased strongly with increasing degree of oxidation (0–≤ 100%). Both increasing temperature (5–75 °C) and pH (5–9) accelerated oxidation, and, consequently, slowed down dissolution kinetics.

## Abstract 2

Surface oxidation decreases the solubility and dissolution kinetics of vivianite particles due to the formation of a passivating amorphous Fe(III)–PO_4_ surface layer and makes P from vivianite less readily bioavailable.

## 1 Introduction

Considering the limited global rock phosphate reserves, their global supply chains, and vital importance for agricultural and other uses,^2^ the European commission considers rock phosphate as a critical raw material (COM/2020/474) and aims to improve circularity and sustainability under its Critical Raw Materials Act (COM/2023/160). In this context, P recycling is key to sustain global food production and to achieve a sustainable circular P economy.^2^ The efficient recovery of the natural ferrous iron phosphate mineral vivianite (Fe_3_(PO_4_)_2_·8H_2_O) in wastewater treatment plants^3−9^ and its potential application as fertilizer^10−17^ may be a promising P recycling strategy.^3,18^ Furthermore, vivianite represents a substantial, previously underestimated P sink in limnic, fluvial, and coastal systems and might therefore be of significance for the global biogeochemical P cycle.^19−21^

Vivianite forms naturally under reducing conditions but is sensitive to oxidation, rendering it metastable under atmospheric conditions. Recently, it was found that the dissolved oxygen concentration influences the crystallization of vivianite.^22^ Therefore, oxidation of vivianite might also impact the dissolution rate and behavior and hence the release of Fe and P. Vivianite dissolution kinetics and mechanisms are well studied in anoxic systems^23,24^ but remain to be elucidated under oxic conditions. Considering that soils are predominantly oxic systems, an understanding of the impact of the oxidation reaction on vivianite dissolution is critical for assessing the potential of vivianite as a P fertilizer.^25−27^ However, as oxidation and dissolution occur concurrently, disentangling rates and mechanisms is challenging.

Pristine vivianite exhibits a monoclinic symmetry with sheets of corner sharing FeO_6_ octahedra and PO_4_ tetrahedra.^28^ These FeO_6_–PO_4_ sheets are connected via weak hydrogen bridges between the H_2_O ligands. The vivianite crystal structure comprises distinguishable monomeric (Fe_A_) and dimeric (Fe_B_) octahedral sites (Figure S1).^29^ Vivianite oxidation is characterized by a distinct, intensifying color change from white to blue to purple, caused by an intervalence charge transfer between the two Fe ions at Fe_B_-sites, facilitated by their very short interatomic distance (2.96 Å).^30−32^ Within the crystal structure, the oxidatively induced positive charge is balanced by the conversion of a H_2_O ligand into an OH^–^ group and the release of a H^+^; eq 1(33,34)
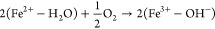
1

The monoclinic symmetry of vivianite is maintained up to 40–50% of Fe-oxidation, and the increasing number of OH^–^ is strengthening the bonds between the layers.^35^ However, at higher oxidation degrees, the monoclinic symmetrical structure of vivianite collapses.^29,36^ The vivianite oxidation sequence has been studied previously^37,38^ and an oxidation pathway from monoclinic vivianite via triclinic metavivianite (Fe^2+^_3–x_Fe^3+^_*x*_ (PO_4_)_2_(OH)_*x*_·(8 – *x*)H_2_O) to amorphous santabarbaraite (Fe^3+^_3_(PO_4_)_2_(OH)_3_·5H_2_O) has been proposed.^37^

The lower symmetry of metavivianite is interpreted as a direct result from the structural collapse of oxidized vivianite.^39^ Metavivianite accommodates Fe(III) contents >47–50% of total Fe (Fe(tot)) with the vague upper limit of oxidation of <100%.^40^ Natural metavivianite samples show a complete oxidation of Fe_A_ sites (100% Fe(III)), while Fe_B_ sites are only partially oxidized (≥20–25% Fe(III)).^40,41^ However, oxidation of vivianite can also result in the direct formation of the oxidation end-product santabarbaraite, hence, skipping the intermediate metavivianite phase.^38^ Under dry, atmospheric conditions, vivianite oxidizes spontaneously and stabilizes at ∼50% (Fe(III)/Fe(tot)), showing no distinguishable change in the X-ray diffractogram despite the increasing Fe(III) content.^33^ Accordingly, no metavivianite forms under these conditions; the conversion to metavivianite was suggested to require higher temperatures.^33^ Chiba et al.^38^ were able to precipitate metavivianite in synthesis solutions at ∼60 °C.

Vivianite oxidation proceeds progressively, and bulk measurements cannot always resolve gradual local changes. Observations from natural samples show a mixture of associated triclinic and monoclinic phases, following a gradient from the exterior to the center of a large crystal.^41^ Vivianite surfaces appear to be extremely sensitive to oxidation and may not even be stable under an inert (N_2_) atmosphere, as supported by XPS measurements.^42^ Surface oxidation occurred by cleaving vivianite along the (010) plane and, hence, breaking the hydrogen bridges.^42^ These observations were explained by an auto-oxidation reaction, previously proposed by Moore^43^ and Hanzel et al.^44^ for the decomposition of crystal water at the vivianite surface in vacuum; eq 2
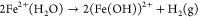
2

In oxic solutions, pristine vivianite oxidizes up to 5–10% within minutes, but the oxidation rate decreases with an increasing degree of oxidation.^33,45^ Solid phase oxidation products may limit diffusion of the reactant (O_2_) and product (H_2_O) (eq 1) and protect the mineral from further oxidation. Accordingly, Hanzel et al.^46^ found a slower oxidation rate for bulk vivianite, compared to the surface.

Although vivianite oxidation has been explored in multiple studies,^29,33,35,36,41,44,46^ its implications for P (and Fe) release under environmentally relevant (oxic) conditions are still elusive. Therefore, the objective of this study was to provide a quantitative and mechanistic understanding of the effect of abiotic vivianite oxidation (O_2_ or H_2_O_2_) on P and Fe release from vivianite. To this end, we performed a series of highly controlled batch and flow-through experiments to examine vivianite solubility and dissolution kinetics as a function of the oxidation degree, as well as vivianite oxidation and dissolution kinetics under oxic conditions as a function of pH and temperature. By combining bulk solution chemistry measurements with (spatially resolved) solid phase characterization including synchrotron-based spectroscopic techniques, we resolved the vivianite transformation into secondary phases and its impact on P and Fe release into solution.

## 2 Materials and Methods

### 2.1 Materials

All chemical reagents used were of ACS grade. Unless otherwise stated, anoxic water, used for all anoxic experiments, was prepared by boiling ultrapure milli-Q water (MQ, 18.2 MΩ·cm^–1^, TOC < 2 ppb), purging it with N_2_ while cooling down (∼4 h) and equilibrating it for ∼24 h with the N_2_ atmosphere inside an anoxic chamber (mBRAUN, Unilab 7185; O_2_ < 1 ppm).

Vivianite was synthesized according to a previously described method.^24^ Briefly, 0.4 M NaH_2_PO_4_·H_2_O and 0.6 M FeCl_2_·4H_2_O solutions were mixed under anoxic conditions. Vivianite precipitation was induced by adding 0.5 M NaOH until the pH reached ∼7. A white precipitate formed readily, which was filtered after ∼24 h (filter paper, 5–8 μm, Whatman) and washed until electroconductivity of the filtrate was <10 μS cm^–1^. The precipitate was dried at room temperature, homogenized with a pestle and mortar, and stored in a desiccator inside the anoxic chamber covered with aluminum foil to minimize light exposure. For consistency and comparison, synthesized vivianite from the same homogenized and thoroughly characterized batch was used in all experiments. The measured specific surface area (SSA) was 1.24 m^2^ g^–1^, and the mean particle diameter was 9.5 ± 6.8 μm. A detailed characterization of this material has been reported previously.^24^

Batches of preoxidized vivianite were prepared by adding aliquots of 3% (w/v) unstabilized H_2_O_2_ solution to rigorously stirred 100 mM synthesized vivianite suspensions to reach predefined oxidation degrees [10, 30, 45, 60, 80, 90, 100% Fe(III)/Fe(tot)]. H_2_O_2_ was added under anoxic conditions to exclude other oxidants, and vivianite was preoxidized >24 h prior to use to ensure a complete reaction.

### 2.2 Experimental Setup

#### 2.2.1 Vivianite Oxidation under Dry Conditions

Dry synthesized vivianite powder (0.05 g) was transferred into 1.5 mL Eppendorf tubes, covered in aluminum foil under atmospheric conditions in a temperature-controlled room (21 ± 1 °C). The experiment started when the samples encountered atmospheric conditions. Samples were taken destructively in triplicate over a period of 49 days by complete dissolution and simultaneously stabilized in 6 M HCl. The Fe(II) and Fe(tot) concentrations were measured to establish the degree of oxidation, as described in Section 2.3.1.

#### 2.2.2 Batch Dissolution Experiment

Batch experiments to investigate oxidation and dissolution rates and the solubility of the synthesized (preoxidized) vivianite under anoxic (inside the N_2_ glovebox) and oxic (atmospheric) conditions were conducted in duplicates at room temperature (21 ± 1 °C) for 18 and 8 days, respectively. Samples were continuously stirred with magnetic stir bars at 300 rpm in 50 mL dark brown glass reaction vessels preventing photo-oxidation. Experiments were initiated by adding vivianite from a 100 mM stock suspension to a solution containing 10 mM pH buffer, resulting in 50 mL of 0.2 mM vivianite suspensions; (as exception, in the experiments with preoxidized vivianite under anoxic conditions, the vivianite concentration was 1.0 mM). Noncomplexing buffers were selected according to the desired pH: *N*,*N*′-diethylpiperazine (DEPP) for pH 5.0 and 9.0, 2-(*N*-morpholino)ethanesulfonic acid (MES) for pH 6.0, 3-(*N*-morpholino)propansulfonic acid (MOPS) for pH 7.0, and piperazine-1,4-bis(propanesulfonic acid) (PIPPS) for pH 8.0.^47^ The pH was adjusted by using 1 M HCl or NaOH, and the ionic strength (IS) was adjusted to 10 mM by using 1 M NaCl, accounting for the contribution of the respective buffers.

For temperature-controlled experiments, batch reactors were placed into a thermostatic water bath (Huber, KISS k6) and continuously stirred with a magnetic stirrer at 300 rpm for 5 days. The actual temperatures of the experimental solutions were directly monitored during the experiment. Temperature-controlled experiments were conducted only at pH 6.0 and ran for 48 h at 5, 25, 50, 65, and 75 °C. The pH was adjusted at 25 °C, accounting for the temperature dependence of the pH buffer.

The pH was monitored throughout the experiments, and samples were collected after set reaction times. Suspension samples were immediately filtered (0.2 μm of CA, Minisart) and stabilized in 1 M HCl to determine the dissolved analyte concentrations. Additionally, unfiltered suspension samples were digested in 6 M HCl to determine the suspension concentrations. All samples were analyzed for total dissolved P and total Fe and Fe(II) concentrations. At the end of each experiment, the remaining solids were retrieved by centrifugation and removal of the supernatant. The retrieved solids were dried under N_2_ atmosphere and stored at room temperature in the anoxic chamber until further analysis.

#### 2.2.3 Continuous Flow Stirred Tank Reactors

To further examine the dissolution and oxidation rates of vivianite, continuous flow stirred tank reactor (CFSTR) experiments were performed under oxic conditions (pH 6.0, 10 mM MES), with initially 1 g of unoxidized vivianite. Effluent samples were collected, immediately stabilized with 1 M HCl, and analyzed for dissolved total Fe, Fe(II) and P concentrations. Additionally, the pH and flow rate were monitored. A detailed description of the reactor design and experimental setup is presented in SI Section 5.1.

Table 1 summarizes all experimental setups and analytical methods.

**Table 1:** Overview of Performed Experiments, Experimental Conditions, and Performed Measurements

experiment	atmosphere	oxidant	(initial) oxidation degree (%)	pH	temperature (°C)	reaction time (d)	analysis
Solid Phase Experiments (Section 3.1)							
vivianite oxidation under dry conditions	air	O_2_	0	-	21 ± 1	49	Ferrozine
bulk characterization of preoxidized vivianite	N_2_	H_2_O_2_	0, 10, 30, 50, 90	∼7	21 ± 1	1	Ferrozine, XRD
spatially resolved characterization of vivianite	air, N_2_	O_2_, H_2_O_2_	0, 10, 20, 30, 100	-, ∼ 7	21 ± 1	1	Ferrozine, STXM
Batch Dissolution Experiment (Section 3.2; 3.3)							
dissolution of preoxidized vivianite	N_2_	H_2_O_2_	0, 10, 30, 45, 60, 80, 100	6.0	21 ± 1	18	Ferrozine, ICP-OES, XRD, SEM-EDX, XAS
dissolution and oxidation of vivianite	air	O_2_	0	6.0	21 ± 1	2	Ferrozine, ICP-OES
dissolution and oxidation of vivianite over temperature	air	O_2_	0	6.0	5, 25, 50, 65, 75 ± 1	5	Ferrozine, ICP-OES, SEM-EDX
dissolution and oxidation of vivianite over pH	air	O_2_	0	5.0, 6.0, 7.0, 8.0, 9.0	21 ± 1	8	Ferrozine, ICP-OES
Flow-Through Dissolution Experiment (Section 3.3.2)							
CFSTR	Air	O_2_	0	6.0	21 ± 1	15	Ferrozine, ICP-OES

### 2.3 Analytical Methods

#### 2.3.1 Wet Chemical Analysis

Total Fe and P concentrations were measured by inductively coupled plasma optical emission spectrometry (ICP-OES; Agilent Technologies, 5110). The Fe redox speciation was determined photometrically within 12 h after sampling by measuring Fe(II) and Fe(tot) concentrations using a Ferrozine-assay,^48^ as adapted by Porsch and Kappler.^49^ For Fe(II), a pH-buffered (pH ∼ 5) Ferrozine solution [0.1% Ferrozine, 50% ammonium acetate (w/v)] was added to acidified samples. For the Fe(tot) analysis, 10% (w/v) hydroxylamine in 1 M HCl was added to the sample to reduce all Fe(III) before adding the Ferrozine solution. Absorbance was measured at 562 nm with a UV–vis spectrophotometer (Tecan, Infinite M Plex). The Fe(III) concentration was calculated as the difference between the measured Fe(tot) and Fe(II) concentration.

#### 2.3.2 Solid Phase Analysis

Initial and retrieved solids at the end of selected experiments were analyzed by X-ray powder diffraction (XRD; Rigaku, Miniflex 600 with Cu Kα radiation (λ = 1.54 Å) equipped with a monochromator), using an anoxic sample holder with zero background silicon base (10–80° 2θ, step size: 0.06 and 0.5° min^–1^), to determine the phase purity and the formation of crystalline secondary phases. A scanning electron microscope (SEM; FEI, Inspect S50) equipped with an energy dispersive X-ray detector (EDX, Apollo XV) was used to visualize particle size and morphology and to estimate the P/Fe ratio of selected particles. SEM samples were prepared by depositing ethanol suspended samples directly on holders with double-sided tape and carbon-coating (Leica, EM SCD 500). During sample preparation, exposure to O_2_ was minimized but could not be completely prevented. SEM images were processed and analyzed using Fiji software.^50^

##### 2.3.2.1 X-ray Absorption Spectroscopy Analysis

For Fe *K*-edge X-ray absorption near edge structure (XANES) spectroscopy, diluted suspension samples were filtered through a cellulose filter paper that was then dried under a N_2_ atmosphere at room temperature and sealed with Kapton tape. Measurements at the XAS beamline at the KIT Light Source (Karlsruhe Institute of Technology, Eggenstein-Leopoldshafen, Germany) were performed in a vacuum chamber at room temperature in transmission mode using gas-filled ionization chambers (30 cm; IC Spec; FMB Oxford Ltd., UK) for the measurement of incident and transmitted photon intensity. For photon energy monochromatization, a double crystal monochromator (DCM) with a pair of Si(111) crystals was used. Photon energy was calibrated by setting the first maximum of the first derivative of the absorption *K*-edge of a metallic Fe foil to 7112 eV, and the *E*_0_ was fixed at 7128.5 eV. Sample and reference spectra were processed and analyzed using the software package Demeter.^51,52^ Normalized XANES spectra were obtained by subtracting a straight line fitted to the data from −100 to −30 eV before the edge and subsequently dividing by a cubic function fitted to the data from 50 to 300 eV above the edge. The experimental spectra were compared with reference spectra,^24,53^ and quantitative phase distribution was obtained by using linear combination fit (LCF) analysis.

##### 2.3.2.2 Scanning Transmission X-ray Microscopy

Synchrotron-based scanning transmission (soft) X-ray microscopy (STXM) is a spectromicroscopic approach that allows XANES spectroscopy on a mapped region with a spatial resolution of ∼40 nm, using a zone plate with 35 nm outermost zone width. STXM was used to spatially resolve oxidation driven changes in Fe redox speciation within single vivianite particles of varying sizes. Five distinct vivianite samples were prepared: (i) unoxidized synthetic (pristine) vivianite (0% ox), (ii) dry vivianite powder exposed to atmospheric conditions for 50 h (10% ox), (iii) a vivianite-MQ suspension, purged with air for 50 h (20% ox), (iv) a vivianite-MQ suspension, oxidized under anoxic conditions with diluted H_2_O_2_ (30% ox), and (v) completely oxidized vivianite using diluted H_2_O_2_ in ∼3-fold stoichiometric (H_2_O_2_/Fe) excess (100% ox). All samples were prepared and kept under anoxic conditions to preserve the desired oxidation degree until analysis. Aqueous suspensions were wet deposited onto Formvar-coated 300-mesh transmission electron microscopy grids (Plano GmbH, SF 162-3) and were let dry under a N_2_ atmosphere. The dry samples were sealed for transport under N_2_ atmosphere and were analyzed using the ambient STXM beamline (10ID-1) at the Canadian Light Source (CLS) under 1/6 atm He.^54^ The Fe 2p image sequences (stacks) of individual particles were acquired with an energy resolution of 0.1 eV in the energy region of interest (700–760 eV) and a pixel spacing of 75 nm over regions of 3–4 by 4–6 μm in size. The acquired data sets were processed using aXis2000 software package,^55^ following the method described in Schaller et al.^56^ In brief, the image stacks were aligned and converted from transmission to linear absorbance using the Lambert–Beer law eq 3
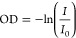
3where OD: optical density; *I*: transmitted photon flux [eV]; *I*_0_: incident photon flux [eV] (from an empty region adjacent to the sample). The resulting image stack was averaged across the entire energy range to obtain the best quality image in the region of interest, and masks were extracted based on 7 OD-ranges. The averaged OD specific spectra were extracted from the image stacks using these masks. Thicker regions (OD > 1.3) were omitted to avoid absorption saturation causing nonlinearity.^57^ The resulting spectra were analyzed by LCF using synthesized (0% ox) and completely oxidized (100% ox) vivianite as reference compounds (normalized to a 1 nm layer) and a sloped background representing nonspecific absorption of non-Fe elements at the Fe 2p edge. The fit was represented in cumulative thickness (nm) and allowed for calculating the respective fractions of Fe(II) and Fe(III). Two independent data sets of each sample were analyzed. For each data set and each thickness range, based on the masks, the fraction of Fe(III) per total Fe (oxidation degree [%]) was calculated and plotted against the cumulative thickness of total absorption (≅ particle thickness).

## 3 Results and Discussion

### 3.1 Characterization of Oxidized Vivianite

#### 3.1.1 Bulk Analysis

When the synthesized vivianite came into contact with O_2_, the color immediately changed from a whitish blue to purple. The purple color intensified as the oxidation progressed until complete oxidation, where the color turned to grayish green (Figure S2). To accelerate oxidation and to reach the desired initial oxidation degrees, vivianite suspensions were preoxidized with H_2_O_2_ under anoxic conditions. Previously, Dormann et al.^36^ investigated the effect of different oxidizing agents (H_2_O_2_, KMnO_4_, O_2_) on the vivianite structure using Mössbauer spectroscopy, XRD, and differential thermal- and thermogravimetric analysis. The transformation from a monoclinic to a triclinic symmetry, as well as the formation of OH- groups, was consistent across the used oxidizing agents, indicating similar structural changes and a common reaction pathway. Accordingly, in terms of structural transformation, oxidation of vivianite by H_2_O_2_ was assumed comparable to atmospheric oxidation, albeit faster.^33,36,41^ The application of H_2_O_2_ resulted in a vigorous reaction with the vivianite. Oxidation was stoichiometric (2:1 Fe/H_2_O_2_) up to oxidation degrees ≤50%; an excess of H_2_O_2_ was needed to reach higher oxidation degrees. The XRD analysis identified vivianite as the only apparent crystalline phase. However, the degree of crystallinity decreased with increasing oxidation degree, as evidenced by the decrease in the peak intensity and by the peak broadening (Figure S3). No differences were observed among the XRD spectra for the different oxidation methods (exposure to air, H_2_O_2_; Figure S4). Oxidation with H_2_O_2_ also did not result in observable morphological changes in the vivianite particles, as evidenced by the SEM images. Also, the P/Fe ratio, determined by SEM-EDX, did not differ significantly (*P* = 0.63 > α = 0.05, *F* = 0.70 < *F*_crit_ = 2.39) among the oxidation degrees (Figure S5). However, a distinct Fe(II) to Fe(III) peak shift from 7126 to 7132 eV in the XANES region was observed, indicating Fe(II) oxidation (Figure S6). LCF analysis indicated that all sample spectra could be satisfactorily (χ^2^ < 0.01) reproduced using only two reference spectra, of unoxidized pristine vivianite and of amorphous Fe(III)–phosphate (Table S1). The fitted fractions matched well with the intended degree of Fe(II) oxidation and were consistent with photometrically determined degrees of oxidation (Figure S7 and Table S1). Thus, we conclude that in our experiments, oxidation led to direct formation of an amorphous Fe(III)–PO_4_ phase, bypassing the intermediate oxidation product metavivianite, as also supported by the XRD analysis (Figure S3). This conclusion is also consistent with previous studies.^38,58−60^ Possibly, rapid oxidation, e.g., resulting from exposure to air, H_2_O_2_, or auto-oxidation by cleavage along 010-plane, does not accommodate the reorganization of the crystal required for the phase transformation from vivianite to metavivianite.^29,33,42^ Furthermore, preoxidation in our experiments was performed at 21 °C, and higher temperatures may be required for metavivianite formation.^33^

#### 3.1.2 Spatially Resolved Characterization

**Figure 1:**
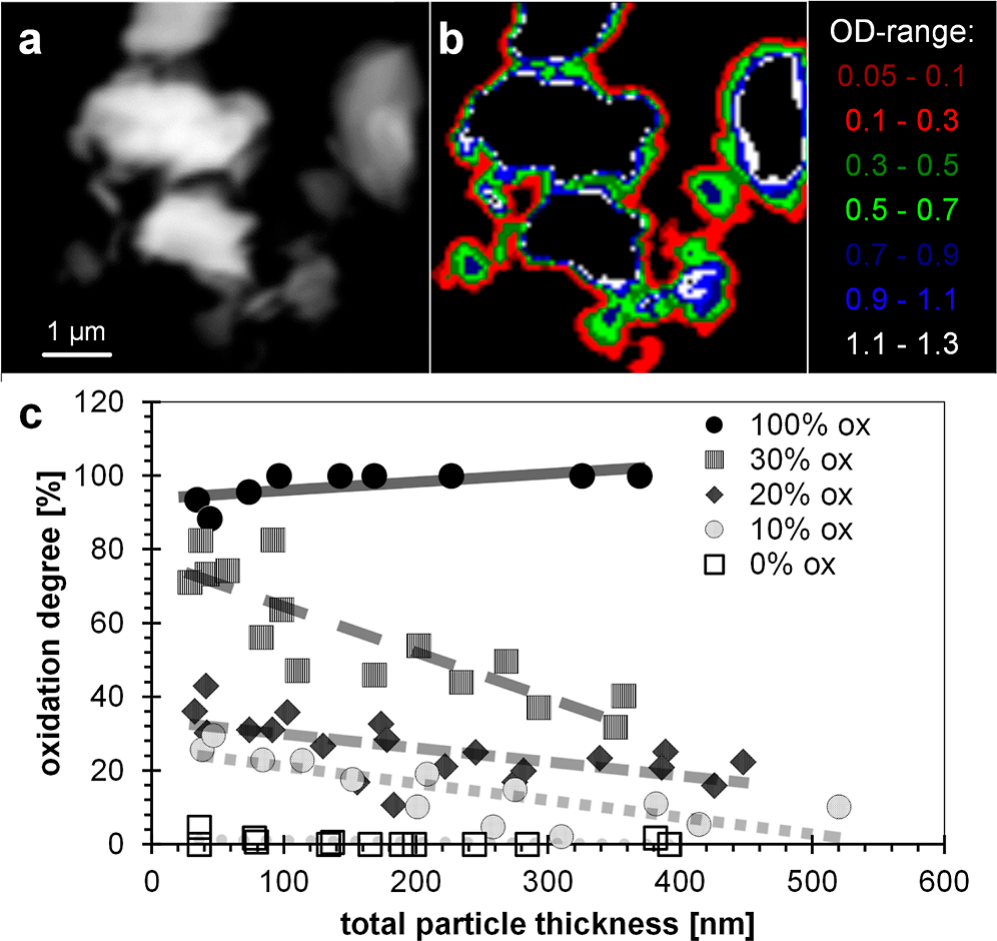
Results from Fe 2p-edge XANES analysis of the surface of vivianite particles oxidized to different degrees. (a) Linear absorbance images and (b) respective thickness masks based on the optical density (OD) that were used to extract spectra of vivianite particles which were oxidized to various degrees (0, 10, 20, 30, 100% of Fe(tot), determined by bulk measurement) by diluted H_2_O_2_ or air. (c) Oxidation degree (i.e., Fe(II)/Fe(tot) ratios) as a function of the cumulative total particle thickness based on the quantitative fits of all acquired data sets. Straight lines represent linear fits for each treatment.

STXM measurements were used to compare the Fe-redox state of thin, and therefore surface dominated, areas of vivianite crystallites to the Fe-redox state of thicker (and therefore more bulk dominated) regions of the same crystallites. In the normalized spectra, a lower spectral signature for Fe(II) was observed in thin, surface-dominated regions (low ODs), compared to thick, bulk-dominated regions (high ODs) (Figure 1). The results were also validated by comparing LCF-derived oxidation degrees from XANES spectra of thick regions with oxidation degrees of bulk vivianite using the Ferrozine-assay after acid digestion. Results obtained from both analyses were in very good agreement (Table S2). Figure 1c shows the determined degree of oxidation as a function of the particle thickness. Each data point represents the results of the LCF fits of the averaged spectra of a cumulative thickness range calculated from the OD (Figure S8). For each sample, at least two regions with several particles have been mapped. Unoxidized synthetic vivianite (0% ox) showed no oxidation, independent of the particle thickness, while the completely oxidized vivianite sample (100% ox) exhibited an oxidation degree of ∼100%. In contrast, for all partially oxidized vivianites (10% ox, 20% ox and 30% ox), the oxidation degree decreased with increasing particle thickness, verifying a core–shell structure. Accordingly, the 10% ox sample showed ∼30% oxidation at the outermost region (∼40 nm) but ∼0% oxidation where the particle was a few 100 nm thick. For the 20% ox and 30% ox treatments, the measurements even on the thickest measured areas (particle thickness ∼400 nm) do not reach ∼0% oxidation. This could be explained by a dilution effect since (i) the contribution of a 0% oxidized core within the mixed signal is smaller at higher oxidation degrees and (ii) thicker particle regions (OD > 1.3), which might be dominated by a 0% oxidized signal had to be omitted because absorption becomes nonlinear and eq 3 is no longer valid. Notably, thicker particle regions (OD > 1.3) account for major parts of the vivianite particles (Figure 1b). In order to illustrate this dilution effect, one may imagine a partially oxidized vivianite particle of thickness *b* in [nm]. The measured degree of oxidation *X* [unitless] of this particle could be represented by a 100% oxidized layer of the thickness *m* in [nm] and a 0% oxidized layer of the thickness (=*b* – *m*). The degree of oxidation of this particle could therefore be described by a simple hyperbolic function; eq 4
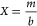
4

Since the vivianite samples were preoxidized, a constant value for *m* may be assumed for each sample (%, 10, 20, 30, and 100% ox). Thus, the proportion of *m* and therefore, also the measured oxidation degree, decrease with increasing total particle thickness (*b*). Using eq 4, and the total particle thickness dependent oxidation degrees (Figure 1c), a constant value for *m* was fitted for each sample (Figure S9). Considering the simplicity of the model, the data could be fitted well, especially at a lower oxidation degree, strongly supporting a core–shell model. Similar core–shell structures have been previously reported for nZVI particles, where oxidation slows down after initial surface oxidation.^61^ However, 100% oxidation was not observed for partially oxidized vivianites, even at the lowest OD range, possibly due to the spatial resolution of ∼40 nm (Figure 1c; the first measurement point is beyond the theoretical thickness of a 100% oxidized shell, vertical red line in Figure S9c, and may therefore be recorded as a mixed signal of oxidized and unoxidized vivianite). Furthermore, the measured oxidation degree decreased with particle thickness more linearly than the simple hyperbolic model would suggest (Figure S9, 30% ox). This may be due to a more complex geometry (the beam diameter may not be approximated as an infinitely small point) and the existence of a partially oxidized diffusive transition zone.

### 3.2 Vivianite Oxidation Kinetics

#### 3.2.1 Oxidation Kinetics of Dry Vivianite under Atmospheric Conditions

After exposure to air at room temperature (21 ± 1 °C), ∼5% of the total Fe in dry vivianite was oxidized within 0.5 h, while it took ∼10 h to reach ∼10% oxidation and ∼50 days to reach ∼20% oxidation (Figure S10). Decreasing oxidation rates with increasing oxidation degrees have been observed previously for dry vivianite under atmospheric conditions.^33^ Hanzel et al.^44^ hypothesized that the accumulation of oxidation products at vivianite surfaces may hinder further oxidation by acting as a diffusion barrier. Accordingly, a logarithmic trend between vivianite oxidation and time, typical for diffusion-controlled (gas–solid) kinetics, was observed (Figure S10). However, the measured oxidation rate in this experiment was slower than previously reported^33^ (Figure S10, with detailed discussion).

#### 3.2.2 Vivianite Oxidation Mechanism and Kinetics in Suspension: Impact of Temperature

**Figure 2:**
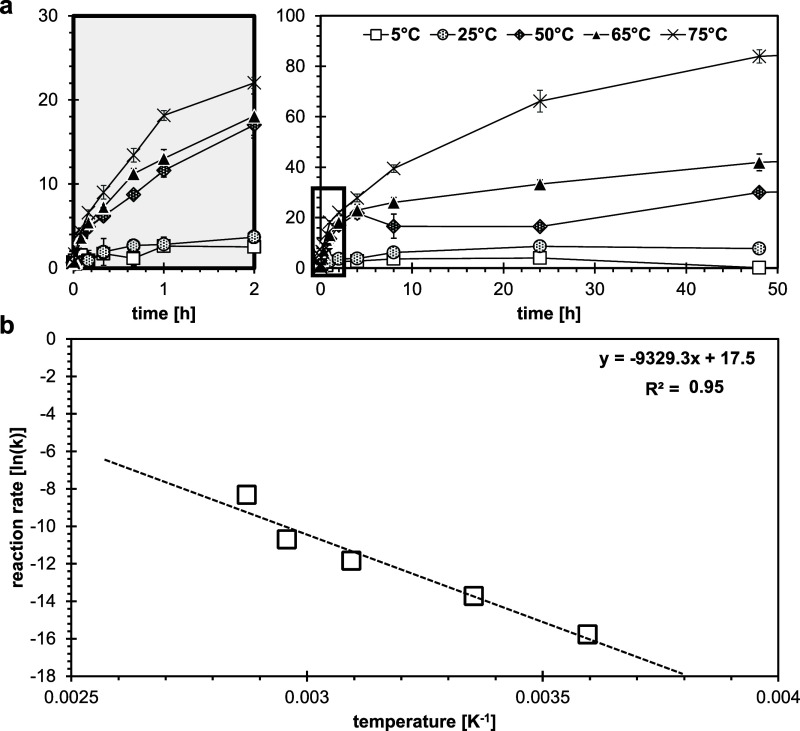
Oxidation kinetics of suspended vivianite (200 μM) over an ambient temperature range (5–75 °C) under atmospheric conditions at pH 6.0 (10 mM MES, IS = 10 mM). (a) Oxidation degree of the solid phase (Fe(III)/Fe(tot)) as a function of time. Error bars indicate deviations between duplicates. For better readability, the initial time interval (0–2 h) has been magnified in the gray outtake. The black frame indicates the magnified area, displayed in the left panel. (b) Arrhenius plot for vivianite oxidation; natural logarithm of the determined oxidation rate coefficients k (h^–1^) based on the 3D diffusion Jander model was plotted as a function of the reciprocal temperature (5–75 °C) in Kelvin (K). Activation energy: *E*_a_ = 77.6 ± 10.3 kJ mol^–1^. To correct for the temperature-dependence of dissolved O_2_ concentration, the determined rates were divided by the dissolved O_2_ concentration at the respective temperature, assuming 100% saturation. For the reaction stoichiometry, it is assumed that 1 O_2_ molecule oxidizes 4 Fe(II).

The mechanism and kinetics of vivianite oxidation in aqueous suspensions under atmospheric conditions were investigated as a function of temperature (5–75 °C) (Figure 2). At all temperatures, oxidation rates were initially relatively fast but slowed down over time. Initial oxidation rates increased with temperature and were much larger at higher temperatures (50, 65, 75 °C) than at lower temperatures (5, 25 °C; Figure 2a). Oxidation kinetics could not be described with simple rate laws, but the reaction appeared to be diffusion controlled (Figure S11), in agreement with the reports by Roldán et al.,^45^ which suggested a parabolic diffusion law where the rate-limiting process is intra- or interparticle diffusion. In diffusion-controlled reactions, the rate of product formation decreases proportionally with the thickness of the product barrier layer.^62^ The kinetic data align well with a shrinking-core model, based on the spectroscopic observations (Figure 1; an extensive discussion of kinetic models is presented in the SI, Section 3.2, Figures S11–S14). To describe the observed oxidation kinetics, several diffusion models were employed considering different geometries (shape factors). The 3D diffusion-Jander model, considering a spherical solid particle, gave the best fit (Figure S12, Table S3). The slopes of the regression lines were used to determine the rate coefficient (*k*) for each temperature. The effect of temperature (*T*) on the rate coefficient can be described with the Arrhenius eq 5
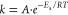
5

By plotting ln(*k*) as a function of *T*^–1^, an Arrhenius plot was obtained (Figure 2b), from which the activation energy *E*_a_ = 77.6 ± 10.3 kJ mol^–1^ and the pre-exponential factor *A* = 0.40 ± 0.06 h^–1^ were determined. An *E*_a_ has not been previously reported for vivianite oxidation, but several studies reported an *E*_a_ for the oxidation of the mixed valence Fe mineral magnetite (Fe^2+^(Fe^3+^)_2_O_4_), ranging between 79.5 and 100 kJ mol^–1^,^63−66^ which is well in line with the found value for vivianite.

### 3.3 Influence of Oxidation on Vivianite Dissolution

#### 3.3.1 Dissolution of Preoxidized Vivianite under O_2_-free Conditions

**Figure 3:**
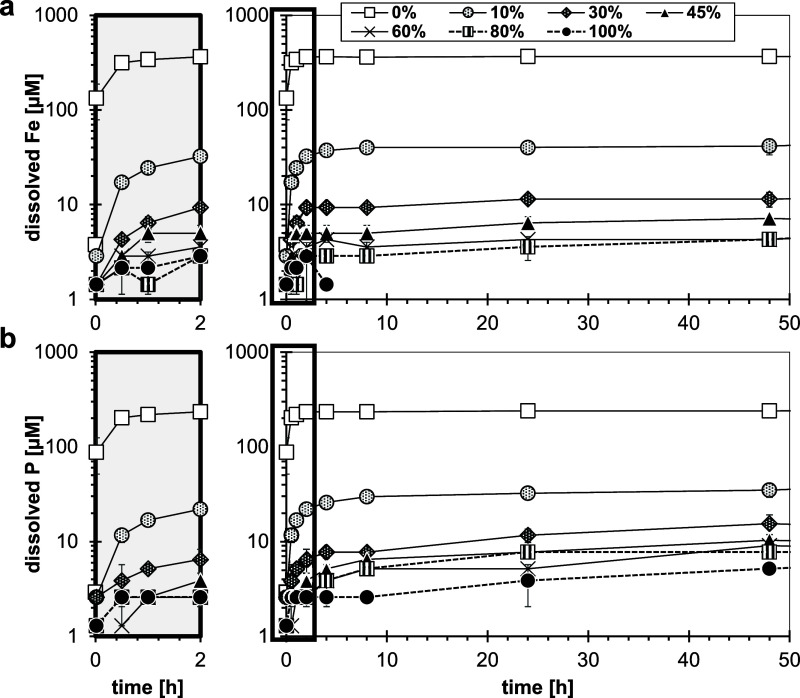
Dissolution of vivianite (1 mM) under anoxic conditions. Vivianite stock suspensions had been preoxidized with diluted H_2_O_2_ solution to various degrees (0–100% Fe(III) of Fe(tot)). The dissolution experiment was performed at pH 6.0 (10 mM MES; IS = 10 mM). Dissolved (a) Fe and (b) P concentrations are presented as a function of time. Error bars indicate deviations between duplicates; LOQ = 1 μM. For better readability, the initial time interval (0–2 h) has been magnified in gray outtakes. The black frames indicate the magnified areas, displayed in the left panels.

To disentangle the oxidation and dissolution reaction, dissolution experiments were performed under anoxic conditions with vivianites that had been preoxidized to desired oxidation degrees. In this setup, the effect of progressive oxidation during vivianite dissolution was eliminated. In all treatments, a comparatively fast initial dissolution stage (up to 2 h) was followed by slower dissolution (Figures 3, S15, and S16). The observed initial dissolution rates (*R*_initial_) decreased strongly with increasing degree of preoxidation, especially from 0 to 10% preoxidation (Figure S16, notice the log-scale). Compared to unoxidized vivianite, preoxidation up to 10 and 30%, lowered the dissolved Fe and P concentrations after 48 h by ∼10-fold and almost 100-fold, respectively. Higher degrees of preoxidation (>30%), however, did not further decrease Fe and P concentrations, which were already close to the limit of quantification (∼10 ppb). With increasing oxidation degree, P was preferentially mobilized: the P/Fe ratios increased from the initial stoichiometric ratio of pristine vivianite ∼0.7 (for up to 10% ox) to ∼2 (for 30–80% ox), and eventually to ∼8 (for 100% ox). The shift of P/Fe ratios may be related to aging of the Fe(III)–PO_4_ phase.^67,68^ Independent of the preoxidation degree, Fe(II) dominated the dissolved Fe speciation (Figure S15). No significant changes in solid phase Fe/P ratio were observed throughout the experiments (Figure S15d), as dissolved concentrations were small (∼10 μM) compared to the total concentrations in suspension (1 mM vivianite). Unoxidized vivianite reached solubility equilibrium within ∼2 h, while 10% ox vivianite, after a fast initial dissolution, only slowly dissolved further, and solubility equilibrium was not reached within 18 days. In contrast, for vivianite with oxidation degrees ≥30%, equilibrium was reached after ∼3 days at much lower concentrations, indicating that a second phase, other than vivianite, controlled the solubility. Since the X-ray diffraction analysis of preoxidized samples established vivianite as the only crystalline phase (Figure S3), solubility may have been controlled by the amorphous, Fe(III)–PO_4_ phase (santabarbaraite) with no distinct XRD signal. The solubility of santabarbaraite has not been determined yet. However, Fe(III)–PO_4_ phases were found to generally have a low solubility; e.g., for Fe_2.5_PO_4_(OH)_4.5_ p*K*_sp_ = 96.7.^69^ Presumably, santabarbaraite covered all particle surfaces, preventing direct contact of the vivianite core with the aqueous phase. At 10% oxidation, however, the oxidized shell was likely heterogeneous and patchy, causing the system to move toward a coupled equilibrium with vivianite and the amorphous Fe(III)–PO_4_ phase, having PO_4_^3–^ as common species. Possibly, during the slow dissolution phase over the 19 days reaction time, abrasion by continuous magnetic stirring may have occurred.^24,70^ This may have contributed to the disintegration of vivianite particles, exposing unoxidized vivianite to direct contact with the aqueous phase. Consequently, the solubility of unoxidized and 10% oxidized vivianite appeared comparable, while the dissolution rates were strongly reduced for the latter. Still, it remains speculative as to why this was not the case for higher oxidation degrees.

#### 3.3.2 Simultaneous Oxidation and Dissolution of Vivianite

Compared to anoxic conditions,^24^ at pH 6, the dissolution rate and the apparent solubility of initially unoxidized vivianite decreased sharply in the presence of O_2_ (Figure S17). The *R*_initial_ decreased by a factor of ∼3 (Table S4), while the mobilized P concentration after 48 h decreased by a factor of ∼7. *R*_initial_ values are difficult to interpret because the addition of unoxidized vivianite to oxic solutions initiated multiple simultaneous reactions, including the dissolution and oxidation of vivianite as well as the oxidation of dissolved Fe(II) and its subsequent precipitation. This becomes more apparent when oxic dissolution is compared in batch reactors (Figure S17) and in a flow-through system (Figure S18). Details on the CFSTR experimental setup and results are presented in SI Section 5.1. While *R*_initial_ in batch experiments was substantially higher (∼1000 times) than the steady-state dissolution rate in the CFSTR setup, dissolution rates became comparable after 8 h (batch_8–49h_: 0.4 ± 0.26 and CFSTR_50–360h_: 0.2 ± 0.06 μM vivianite m^–2^ h^–1^, Figure S17, Tabel S4); rates were calculated only from dissolved P concentrations and were normalized to vivianite stoichiometry. The comparability of results from the two experimental setups supports that solution saturation was not reached and that steady state dissolution can be assumed for the CFSTR setup. However, the final oxidation degree for vivianite in the CFSTR experiment was ∼35% after 15 days (Figure S7, Table S1; LCF fit). Comparing the steady state dissolution rate (CFSTR) to the *R*_initial_ of 30% preoxidized vivianite under anoxic conditions (batch; 3.9 μM vivianite m^–2^ h^–1^), the latter was ∼10 times higher than the dissolution rate in the CFSTR setup. Since the calculated progressive oxidation of mobilized Fe(II) (and concomitant coprecipitation of P) is negligible at the experimental conditions [0.1 μM Fe(II) h^–1^, pH 6^23,71^], the different dissolution rates may be explained by the lower oxidation degree of vivianite in the batch experiment (Figure S7), the initial existence of high reactive sites, and possible Fe(II) catalyzed dissolution of Fe(III)-phases under anoxic conditions.^72^

##### 3.3.2.1 Temperature Dependence of Oxic Dissolution of Initially Unoxidized Vivianite

The temperature-dependence of oxic vivianite dissolution at pH 6 was studied over the temperature range 5–75 °C (Figure S19). Fast *R*_initial_ values were observed, which generally decreased with increasing temperature (Figure S19a). At lower temperatures (5 and 25 °C), P concentrations reached a steady state after 10–20 min, while at higher temperatures (50, 65, and 75 °C), gradual P mobilization persisted over time. As a result, total released P concentrations at higher temperatures exceeded those at lower temperatures after ∼48 h (Figure S19a). Furthermore, with increasing temperature, dissolution became increasingly nonstoichiometric (P/Fe ratio increased from 0.6 at 5 °C to 4.5 at 75 °C). Using SEM-EDX measurements, significant differences (*F* = 3.29 > *F*_crit_ = 2.60; *P* = 0.02 < α = 0.05) in the P/Fe ratios between the solid samples at 75 °C and samples from lower temperatures were observed (Figure S20). The SEM images also showed morphological changes related to disintegration of vivianite particles at 75 °C. Therefore, the maximum temperature at which vivianite is stable under oxic conditions appears to be between 65 and 75 °C. Accordingly, the oxidation degree at 75 °C was very high (>80% after 48 h) suggesting an almost complete transformation of vivianite to amorphous Fe(III)–PO_4_ in our study.

##### 3.3.2.2 pH Dependence of Oxic Dissolution of Initially Unoxidized Vivianite

**Figure 4:**
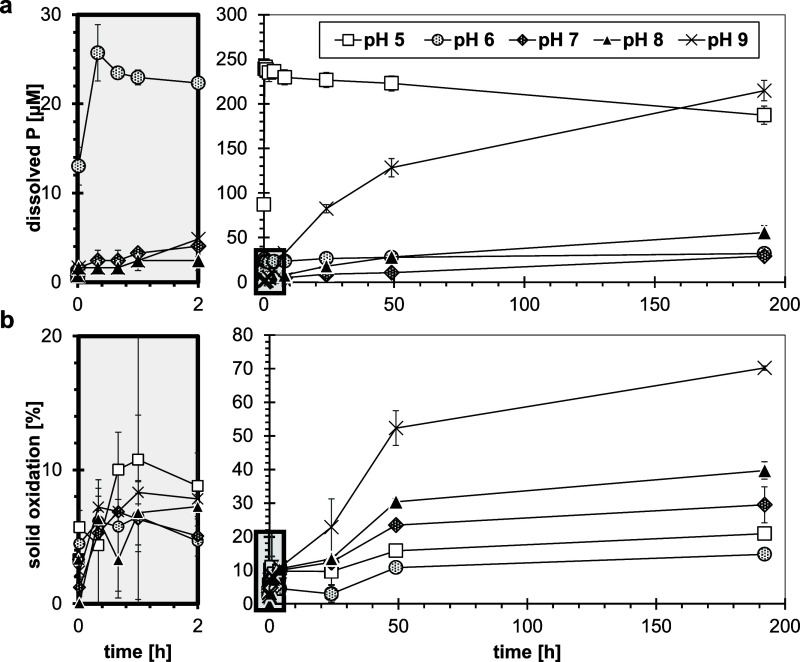
Dissolution of vivianite (200 μM) over an environmentally relevant pH range (5–9) under atmospheric conditions in pH-buffered solutions (IS = 10 mM). (a) Dissolved P concentration and (b) oxidation degree of the solid phase (ratio Fe(III)/Fe(tot)) as a function of time. Error bars indicate deviations between duplicates. For better readability, the initial time intervals (0–2 h) were magnified (gray outtakes). Black frames indicate the magnified areas, displayed in the left panels.

Both vivianite dissolution and Fe-oxidation are pH-dependent reactions. It has previously been shown that under anoxic conditions, dissolution kinetics of pristine vivianite strongly decrease with increasing solution pH.^24^ Also, under oxic conditions, the *R*_initial_ values were higher at low pH values (Figure 4a). Fast, stoichiometric initial dissolution was observed at pH 5 and 6. Dissolved P concentrations reached a maximum after ∼20 min, after which they slowly decreased, possibly due to P association with secondary Fe(III)-precipitates formed upon oxidation of dissolved Fe(II).

Surface protonation can catalyze dissolution reactions.^73^ For vivianite, protonation of PO_4_ at the surface results in the weakening of structural Fe-(PO_4_) bonds due to polarization, promoting Fe detachment.^23^ In contrast, deprotonation at neutral to alkaline pH (7, 8 and 9) did not noticeably increase *R*_initial_ (Figure 4a). However, in this pH range, the dissolved P concentrations continued to gradually increase throughout the experiment, and eventually, the mobilized P concentrations were larger at pH 9 than at lower pH values. At alkaline pH, dissolution was incongruent with dissolved Fe concentrations remaining around the quantification limit (Figure S21). Furthermore, the high oxidation degree (∼70%) and high dissolved P fraction (50%) indicate that the vivianite structure was no longer completely intact at pH 9, which is the pH stability limit of vivianite.^74^ A secondary amorphous Fe(III)–PO_4_ must have formed with a substantially lower P/Fe ratio.

A strong pH dependence of the oxidation kinetics of dissolved Fe(II) by dissolved O_2_ has previously been observed and was attributed to the much higher reactivity of hydrolyzed Fe(II) species.^75−77^ Additionally, once hydrous Fe(III) oxides form as secondary oxidation products, they autocatalytically enhance Fe(II) oxidation rates.^75^ In contrast, our observed oxidation rates were initially comparable among the pH values (Figure 4b). Only at longer time scales (>2 h), the oxidation rate increased with pH, with exception of pH 6. Because P mobilization was slow compared to anoxic conditions and the P mobilization and oxidation rates were decoupled (Figure 4), it can be assumed that Fe(II) is mostly oxidized in the mineral structure and not in solution. Nevertheless, higher pH may promote structural Fe-oxidation. According to density-functional theory computations, the most stable vivianite surface (010-plane) is H_2_O-terminated,^78^ while a completely hydroxylated surface is a highly unstable configuration. The pH-dependent deprotonation of the surface H_2_O molecules of vivianite may destabilize the surface and promote Fe-oxidation.

The oxidation of structural Fe coupled with the precipitation of a secondary amorphous Fe(III)–PO_4_ phase and concomitant preferential P release may cause the observed nonstoichiometric dissolution, in accordance with previous observations.^23^

## 4 Environmental Implications

Vivianite surfaces are readily oxidized under oxic conditions. Our results show that at circumneutral pH and ambient temperatures, vivianite oxidation does not lead to disintegration but to a stabilization of the mineral against dissolution due to the formation of a core–shell structure. This structure consists of a pristine vivianite core and a passivating oxidized amorphous Fe(III)–PO_4_ surface layer, which decreases the P and Fe availability from vivianite substantially.

These findings have strong implications for the role and application of vivianite as a sink or source of P since in many natural aquatic environments, redox fluctuations, including oxidation events, are common and may promote the suitability of vivianite for P burial.

However, also during recovery and application of vivianite as a recycled P fertilizer, oxidation will be inevitable, and the associated decrease in dissolution kinetics may prevent a sufficient P supply to plants. Still, slow but continuous P release was observed from vivianite, particularly at high pH and elevated temperatures (Figures 2a and 4a). Therefore, vivianite might be suitable as long-term, and therefore low maintenance, P fertilizer (alike struvite^79^), which additionally may prevent unintended P losses to surface waters and consequent eutrophication. Under reducing conditions, e.g., in rice paddy soils, vivianite may be a fast-acting fertilizer, even if partially oxidized, since the amorphous Fe(III)–PO_4_ shell may be prone to reductive dissolution, leading to Fe and P release.

Furthermore, the high P/Fe ratio of the amorphous oxidized shell prevents the formation of more crystalline and recalcitrant Fe(III) phases, such as lepidocrocite, hematite, or goethite.^67,80−82^ The amorphous Fe(III)–PO_4_ shell may serve as a relatively available Fe source for plants and microorganism. This may also explain the suitability of vivianite as an Fe fertilizer, as reported in previous studies.^12−15^

In conclusion, due to its high redox reactivity (oxidation, possible rereduction), vivianite can act as a dynamic P source or sink. Its long-term stability, role in environmental systems, and significance for the global P biogeochemical cycle remain to be determined. Our study provides a first quantitative and mechanistic understanding of vivianite dissolution under oxic conditions, exploring the potential of vivianite as a sustainable fertilizer. However, additional studies are required to further test this potential, including the concerns of incorporation of contaminants from wastewaters into the vivianite structure. In follow up studies, we will address these concerns and explore the ability of biogenic ligands to enhance vivianite dissolution under oxic conditions to better understand the behavior of vivianite in more complex (environmental) systems such as soils.

## Supporting Information Available

Vivianite crystal structure; vivianite color change; XRD diffractograms; SEM-EDX images and analysis of H_2_O_2_ oxidized vivianite; XANES spectra and LCF results; STXM spectra and LCF results; hyperbolic fit of oxidation degree as a function of particle thickness; vivianite oxidation under dry oxic conditions and discussion; discussion of vivianite oxidation kinetics; comparison of different kinetic models; linearization of vivianite oxidation considering diffusion kinetics; determination of a diffusion constant; Arrhenius plot; dissolved Fe(II), Fe(tot), P concentration, and solid oxidation degree of H_2_O_2_ preoxidized vivianite under anoxic conditions; dissolution rates of H_2_O_2_ preoxidized vivianite under anoxic conditions; comparison of dissolved P concentrations under anoxic and oxic conditions; discussion and results of CFSTR experiments; dissolved P and Fe concentration at different temperatures under oxic conditions; SEM-EDX images and analysis of oxidized vivianite after dissolution at different temperatures under oxic conditions; and dissolved Fe concentration over pH (5–9) under oxic conditions (PDF)
