# Supplementary material for: Effect of Oxidation on Vivianite Dissolution Rates and Mechanism
Source: Environ Sci Technol. 2024 Aug 16;58(34):15321–32. doi: 10.1021/acs.est.4c04809 (PMC11360369; doi:10.1021/acs.est.4c04809)
Supplement: Supplementary file 1 — es4c04809_si_001.pdf [file es4c04809_si_001.pdf]

Effect of Oxidation on Vivianite Dissolution Rates and Mechanism

Rouven Metz[1], Naresh Kumar[2*], Walter D.C. Schenkeveld[2*], Martin Obst[3], Andreas Voegelin[4], Stefan Mangold[5], Stephan M. Kraemer[1]

* corresponding author: naresh.kumar@wur.nl ORCiD- 0000-0002-8593-5758

walter.schenkeveld@wur.nl

[1]Centre for Microbiology and Environmental Systems Science, Department for Environmental Geosciences, University of Vienna, Josef-Holaubek-Platz 2, 1090 Vienna, Austria

[2]Soil Chemistry, Wageningen University and Research, Droevendaalsesteeg 3, 6708 PB Wageningen, The Netherlands

[3]Experimental Biogeochemistry, BayCEER, University of Bayreuth, Dr. Hans-Frisch-Straße 1-3, 95448 Bayreuth, Germany

[4]Eawag, Swiss Federal Institute of Aquatic Science and Technology, Department of Water Resources and Drinking Water, Ueberlandstrasse 133, CH-8600 Duebendorf, Switzerland

[5]Karlsruhe Institute of Technology, Institute for Photon Science and Synchrotron Radiation, Hermann-von-Helmholtz Platz 1, D-76344 Eggenstein-Leopoldshafen, Germany

Supporting Information

30 pages
3 text section
21 figures
7 equations
4 tables

Table of Contents

Table of Figures 3
1. Vivianite crystal structure 5
2. Characterization of oxidized vivianites 6
2.1. Bulk analysis of oxidized vivianite 6
2.1.1. Color change of oxidized vivianite 6
2.1.2. XRD diffractograms of pristine and pre-oxidized vivianite 7
2.1.3. SEM images and EDX analysis of pristine and pre-oxidized vivianite particles 8
2.1.4. XANES spectra, references and LCF fits of pristine and pre-oxidized vivianite 11
2.2. Spatial resolved characterization of vivianite 12
3. Vivianite oxidation kinetics 14
3.1. Oxidation of dry vivianite under oxic conditions 14
3.2. Vivianite oxidation mechanism and kinetics in suspension 15
4. Dissolution of pre-oxidized vivianite under anoxic conditions 22
5. Vivianite dissolution under oxic conditions 23
5.1. Flow-through dissolution experiment under oxic conditions 23
5.2. The temperature dependence of vivianite dissolution under oxic conditions 25
5.2.1. SEM images and EDX analysis vivianite particles after dissolution under oxic conditions at different temperatures 26
5.3. pH dependence of vivianite dissolution under oxic conditions 28
6. References 29

Table of Figures

Figure S1: Crystal structure of vivianite....5

Figure S2: Color change of vivianite with increasing oxidation degree....6

Figure S3: XRD diffractograms for synthesized pristine vivianite (Synthetic vivianite) and vivianites which were oxidized with diluted H_2O_2 to various degrees (10, 30, 50, 90%). 7

Figure S4: X-ray diffractogram of a reference spectrum of vivianite (Ref. Vivianite; Capitelli, et al. [2]), synthesized vivianite in this study (Synth. Viv (anoxic)), 20 % oxidized vivianite - 24 h in an aerated solution (20% oxidized (air)), and 10 % oxidized vivianite using a diluted H_2O_2 solution (10 % oxidized (H_2O_2)).. 8

Figure S5: SEM-EDX images of H_2O_2 oxidized vivianites and box and whisker plot shows the P:Fe ratio determined by EDX for the spots, indicated in the SEM images. 10

Figure S6: Linear combination fits and XANES spectra of oxidized vivianites using two reference spectra.. 11

Figure S7: Results from the linear combination fitting (LCF) of XANES spectra of oxidized vivianites. 12

Figure S8: Two representative Fe2p edge XANES spectra of STXM measurement (averaged for OD: 0.1 – 0.9) of pristine (0% ox) and complete oxidized (100% ox) vivianite....13

Figure S9: Oxidation degree as function of particle thickness of the 0%, 10%, 20% and 30% artificially oxidized vivianite STXM samples....14

Figure S10: Oxidation degree (Fe(II)/Fe(tot)) of synthesized dry vivianite powder under atmospheric conditions (21 ± 1 °C) in the dark as a function of time....15

Figure S11: Comparison of experimental vivianite oxidation data at various temperatures (5 – 75 °C) as a function of time (symbols) with fits using different models (lines).. 17

Figure S12: Linearization of experimental vivianite oxidation data at various temperatures (5 – 75 °C) according to different diffusion models as a function of time. 18

Figure S13: Linearization of temperature dependent oxidation of vivianite. 20

Figure S14: Oxidation kinetics of suspended vivianite (200 µM) over an ambient temperature range (5-75 °C) under atmospheric conditions at pH 6.0 (10 mM MES, IS=10 mM). Arrhenius-type plot for vivianite oxidation. 21

Figure S15: Dissolution of vivianite (1 mM) under anoxic conditions in buffered solution (IS=10 mM) at pH 6.0 which was previously oxidized to a certain degree (0-100% of Fe(tot)) with diluted H_2O_2 solution....22

Figure S16: Dissolution rate (R) and initial dissolution rate ($R_{initial}$) of vivianite, where stock suspensions had been oxidized to a certain degree with diluted H_2O_2. 22

Figure S17: Comparison of initially pristine vivianite dissolution under anoxic (open squares) and oxic (filled circles) conditions at pH6.0 (10 mM MES; IS=10 at room temperature 21 °C). 23

Figure S18: Continuous flow stirred tank reactors (CFSTR) dissolution experiment of 0.1 g vivianite (solid-to-solution ratio of 1.11 g L^{-1}) under oxic conditions. 24
Figure S19: Dissolved a) P and b) Fe concentration as a function of time resulting from vivianite dissolution (200 µM) at various temperatures (5-75°C) under atmospheric conditions at pH 6 (10 mM MES; IS=10 mM). 25
Figure S20: SEM-EDX images of oxidized vivianites at different temperature; a) 5°C, b) 25°C, c) 50°C, d) 65°C, e) 75°C. Box and whisker plot shows P:Fe ratios determined by EDX for the spots, indicated in the SEM images. 27
Figure S21: Dissolved Fe concentrations as a function of time upon vivianite dissolution (200 µM) over an environmentally relevant pH range (5-9) under atmospheric (oxic) conditions in buffered solutions (IS=10 mM). 28

1. Vivianite crystal structure

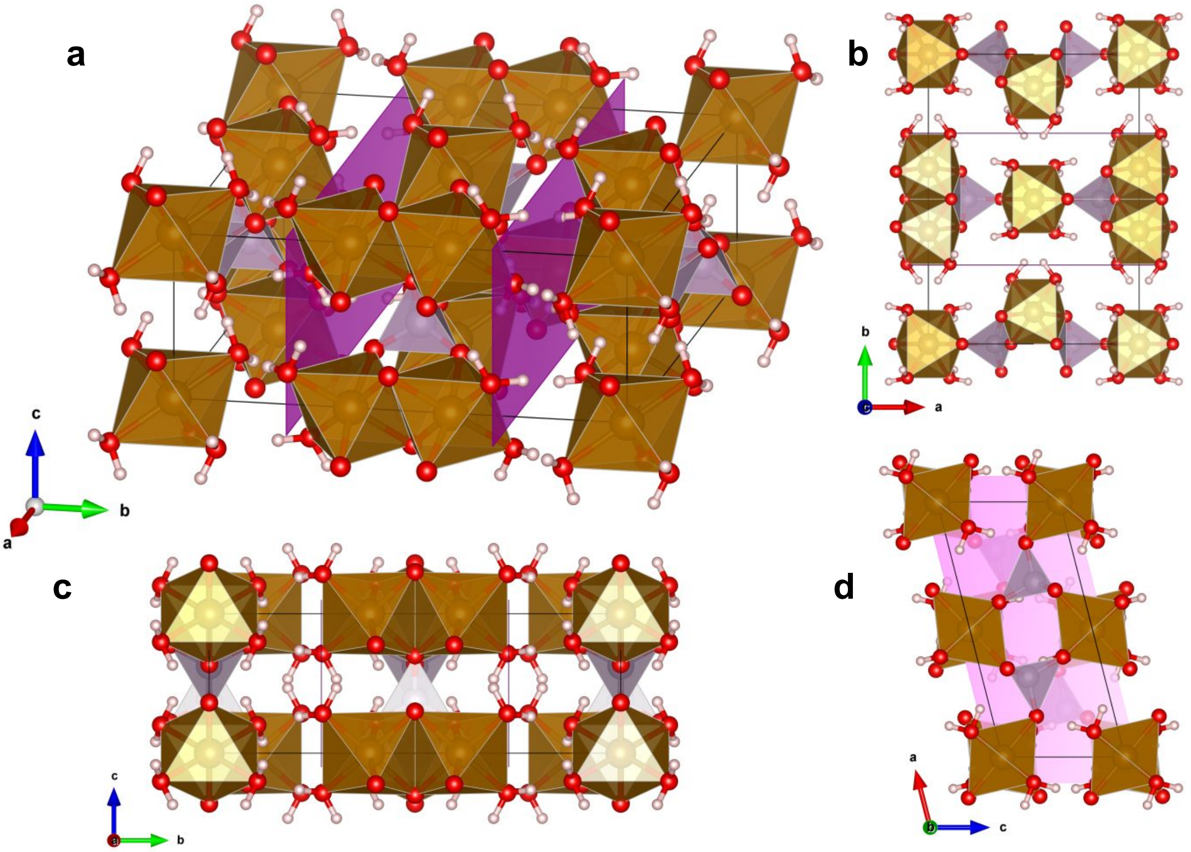

Figure S1: Crystal structure of vivianite viewed in a) standard orientation, b) a-b plane illustrating single $Fe(II)_A$ *and double, edge sharing* $Fe(II)_B$ *octahedra, c) b-c plane and d) c-a plane. In brown, Fe octahedra, and purple:* PO_4 *tetrahedra. Red: oxygen atoms and whitish: Hydrogen atoms. Hydrogen bridge bonds were omitted for clarity. The unit cell is indicated by black lines, and the (010)-plane, responsible for a.o. the perfect cleavage of vivianite, is illustrated in pink. The crystal structure was drawn using the VESTA software*[1] *based on the crystallographic information by Capitelli, et al.* [2]*, adapted from Metz, et al.* [3]

2. Characterization of oxidized vivianites

2.1. Bulk analysis of oxidized vivianite

2.1.1. Color change of oxidized vivianite

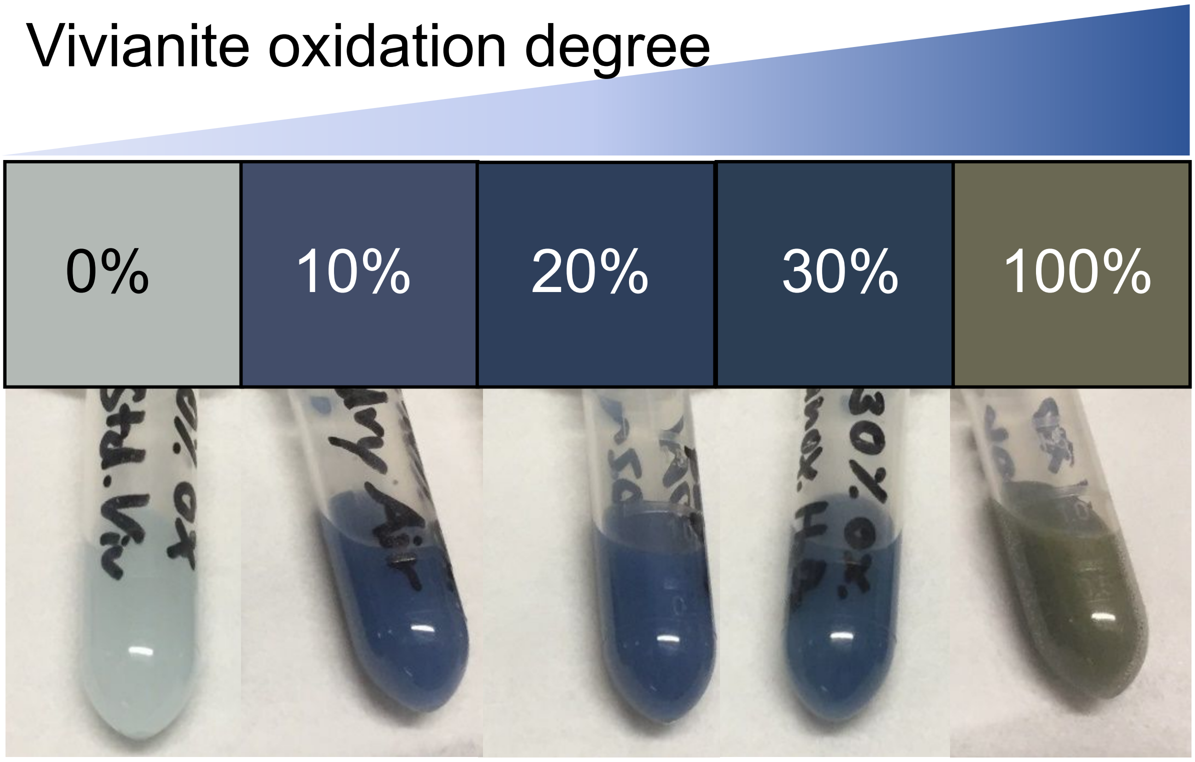

Figure S2: Color change of vivianite with increasing oxidation degree

2.1.2. XRD diffractograms of pristine and pre-oxidized vivianite

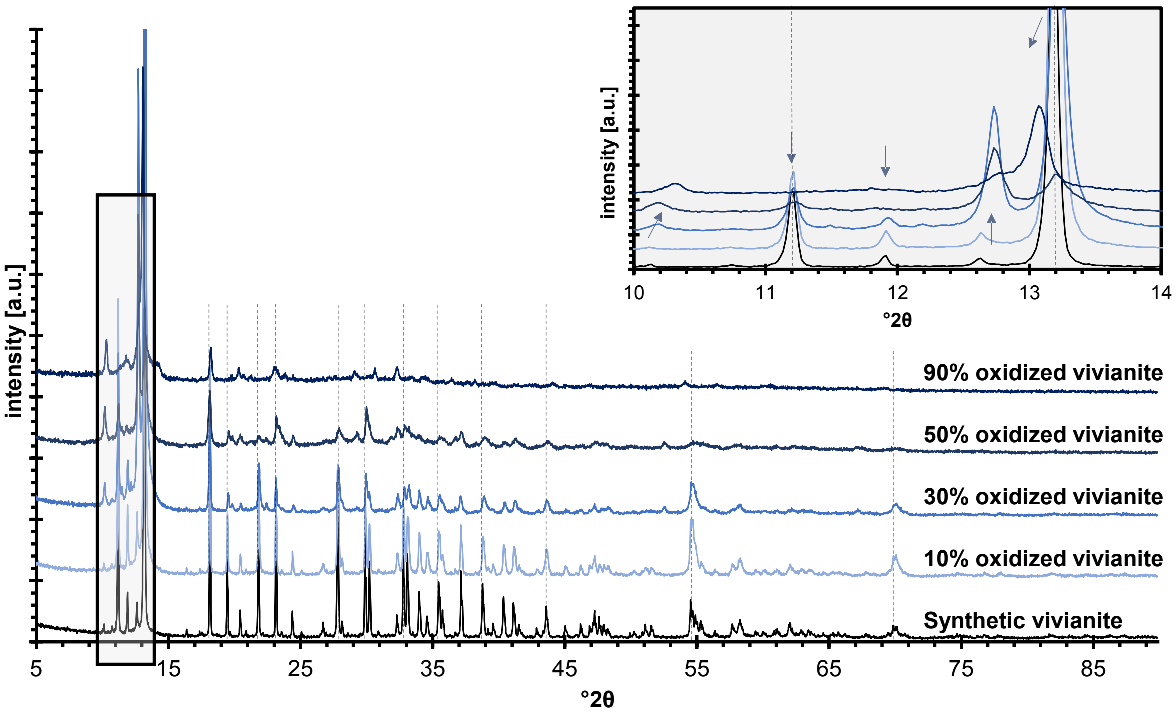

Figure S3: XRD diffractograms for synthesized pristine vivianite (Synthetic vivianite) and vivianites which were oxidized with diluted H_2O_2 to various degrees (10, 30, 50, 90%). Grey dotted lines indicate some main peaks of vivianite. Outtake with grey background magnifies for a better readability the area of 10 – 14 °2θ, indicated by the overlayed black box in the entire diffractogram. The blue arrows point out the major peak changes within the magnified area. Despite substantial changes within the diffractogram, vivianite was the only detected crystalline phase, and emerging peaks could not be matched by other phases.

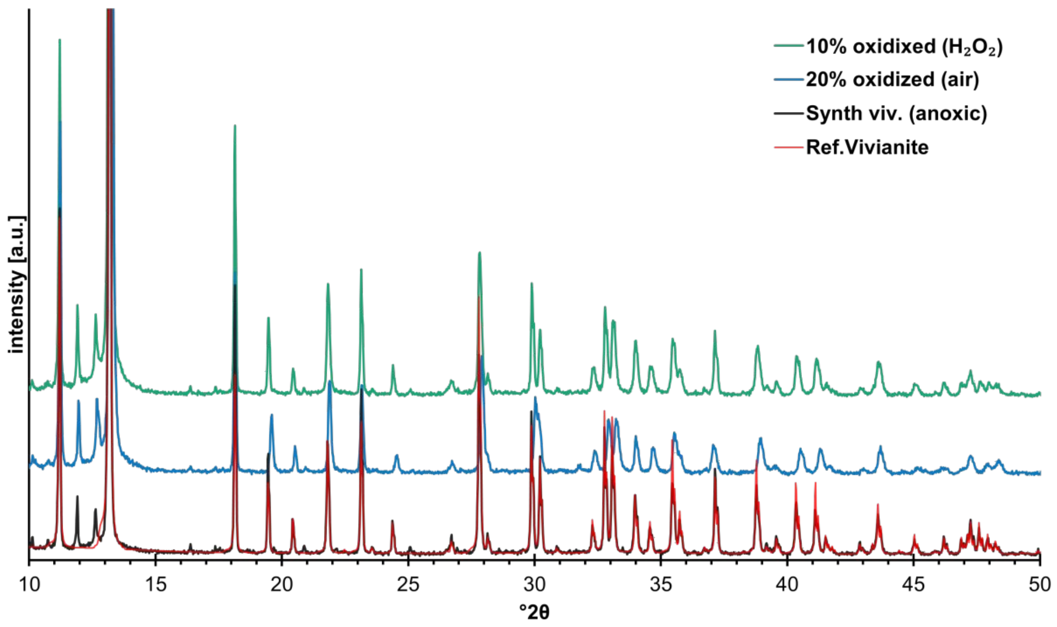

Figure S4: X-ray diffractogram of a reference spectrum of vivianite (Ref. Vivianite; Capitelli, et al. [2]), synthesized vivianite in this study (Synth. Viv (anoxic)), 20 % oxidized vivianite - 24 h in an aerated solution (20% oxidized (air)), and 10 % oxidized vivianite using a diluted H_2O_2 solution (10 % oxidized (H_2O_2)). In all spectra, vivianite was the only detected solid phase. The peaks at 11.9 and 12.6° 2θ could not be assigned to any phase but were also detected in previous studies (Kubeneck, et al. [4]). No differences in phase transformation were observed among the different oxidation methods used in accordance with previous findings (Dormann, et al. [5]).

2.1.3. SEM images and EDX analysis of pristine and pre-oxidized vivianite particles

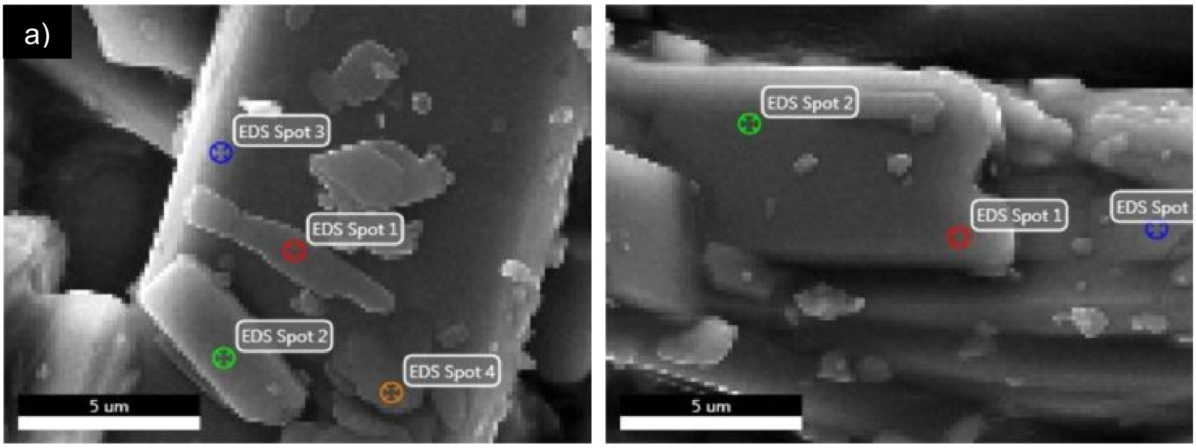

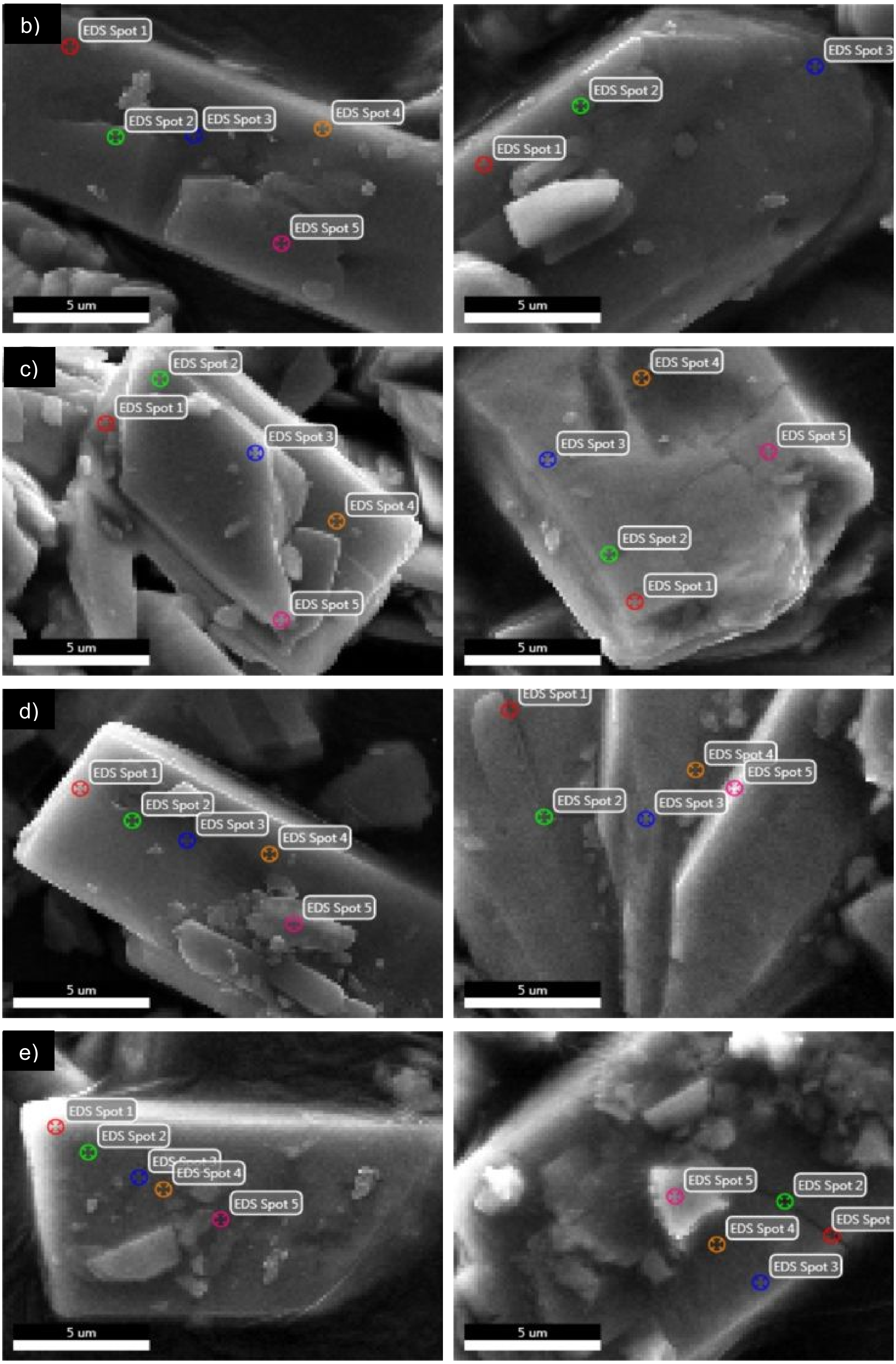
b)
EDS Spot 1
EDS Spot 2
EDS Spot 3
EDS Spot 4
EDS Spot 5
5 um
EDS Spot 3
EDS Spot 2
EDS Spot 1
5 um
c)
EDS Spot 2
EDS Spot 1
EDS Spot 3
EDS Spot 4
EDS Spot 5
5 um
EDS Spot 4
EDS Spot 3
EDS Spot 5
EDS Spot 2
EDS Spot 1
5 um
d)
EDS Spot 1
EDS Spot 2
EDS Spot 3
EDS Spot 4
EDS Spot 5
5 um
EDS Spot 1
EDS Spot 4
EDS Spot 5
EDS Spot 2
EDS Spot 3
5 um
e)
EDS Spot 1
EDS Spot 2
EDS Spot 4
EDS Spot 5
5 um
EDS Spot 5
EDS Spot 2
EDS Spot
EDS Spot 4
EDS Spot 3
5 um

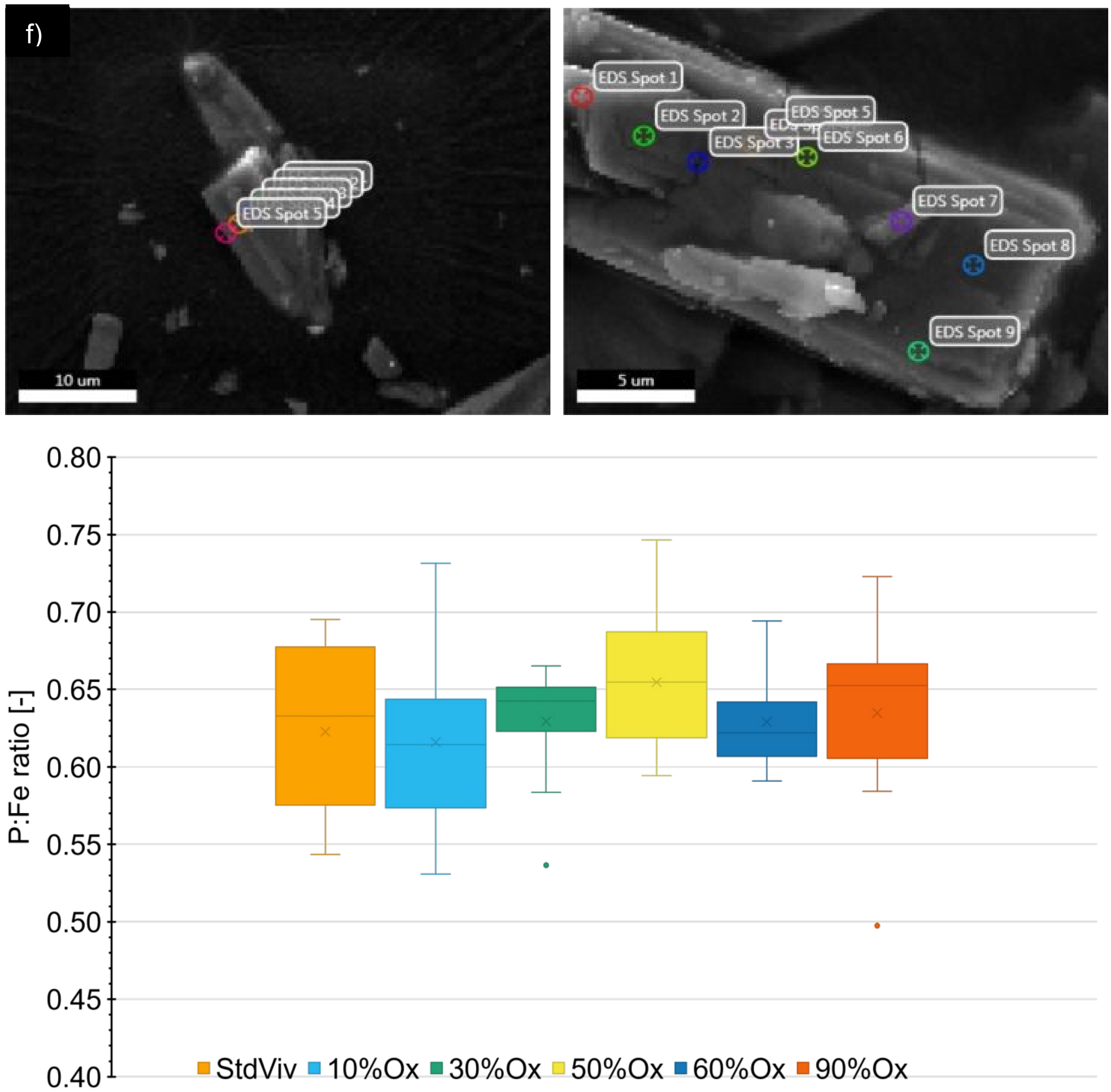

Figure S5: SEM-EDX images of H_2O_2 oxidized vivianites. a) Synthesized vivianite (StdViv), b) 10% oxidized vivianite, c) 30% oxidized vivianite, d) 50% oxidized vivianite, e) 60% oxidized vivianite, f) 90% oxidized vivianite. Box and whisker plot shows the P:Fe ratio determined by EDX for the spots, indicated in the SEM images.

2.1.4. XANES spectra, references and LCF fits of pristine and pre-oxidized vivianite

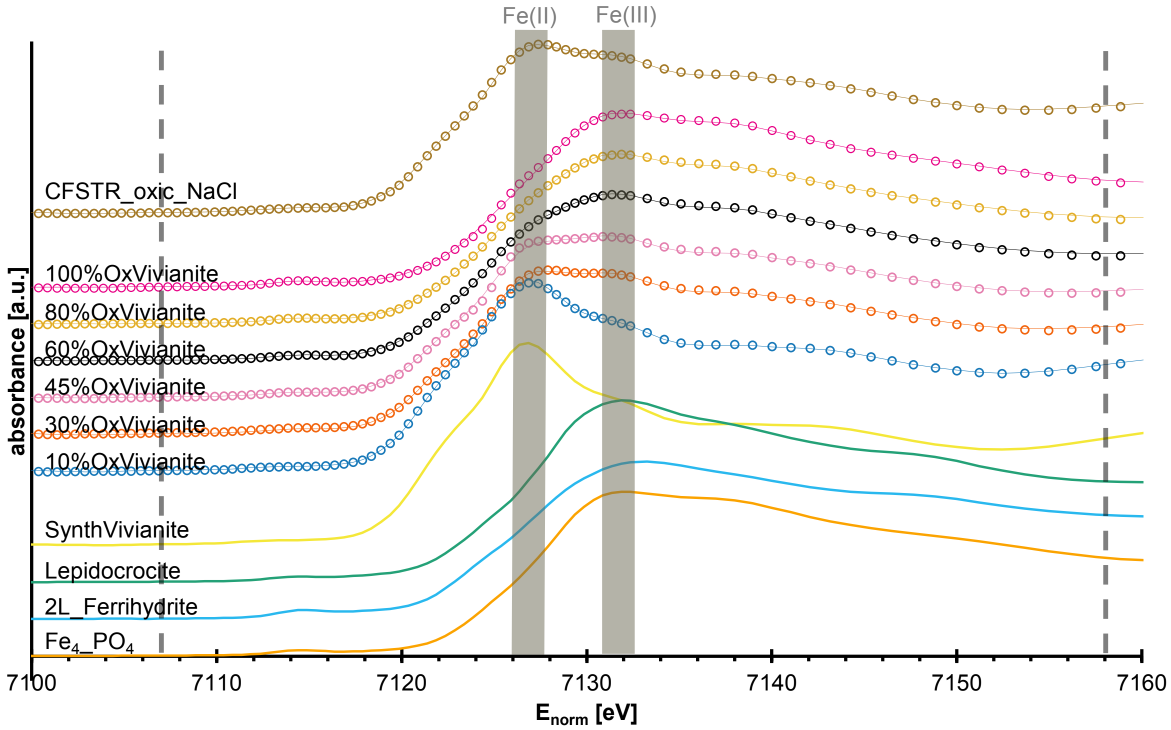

Figure S6: Linear combination fits and XANES spectra of oxidized vivianites using two reference spectra. Dotted black vertical lines indicate the energy range considered for fitting, and the grey vertical lines indicate peak position of Fe(II) and Fe(III).

Table S1: Results from the linear combination fitting (LCF) of XANES spectra of oxidized vivianites. Only two reference spectra (amorphous Fe(III) phosphate: Fe4_PO4 [6]) and synthesized vivianite: SynthVivianite[3]) were necessary to describe all spectral features and without increasing the R-factor significantly.

FITTING RANGE: E_{MIN} -20; E_{MAX}: 60

	Fe4_PO4*	SynthVivianite	r-factor	Chi-square
**10%**	0.111	0.898	0.000273	0.00546
**30%**	0.443	0.603	0.000326	0.00668
**45%**	0.519	0.524	0.000443	0.00928
**60%**	0.692	0.367	0.000201	0.00451
**80%**	0.805	0.263	0.000184	0.00435
**100%**	0.954	0.122	0.000301	0.00774
**CFSTR_NACL**	0.362	0.672	0.000195	0.00397

**For detailed characterization and synthesis of Fe4_PO4 see Voegelin, et al. [6]. In short; 40 ml of 1 M $Fe(III)(NO_3)_3{\cdot}9H_2O$ solution were rapidly added under vigorous stirring to 200 mL pH-adjusted (9.3) 0.5 M $(NH_4)_2HPO_4$ solution.*

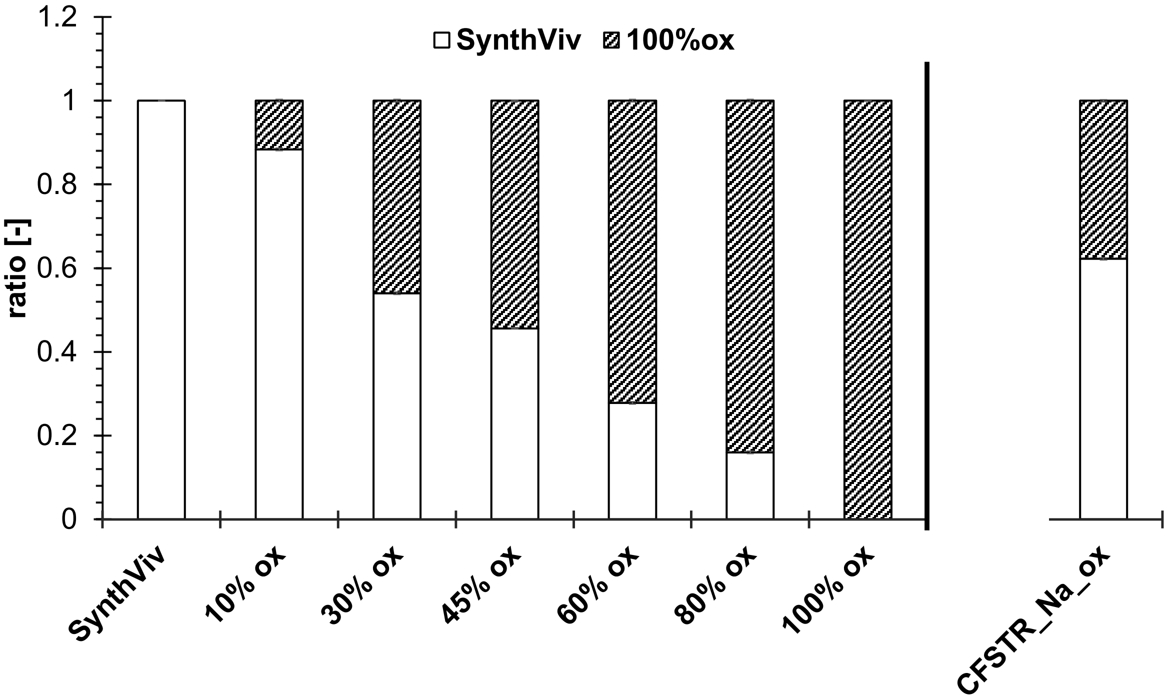

Figure S7: Results from the linear combination fitting (LCF) of XANES spectra of oxidized vivianites. Only two reference spectra (amorphous Fe(III) phosphate: Fe4_PO4 [6] and synthesized vivianite: SynthViv [3]) were necessary to describe all spectral features and without increasing the R-factor significantly.

2.2. Spatial resolved characterization of vivianite

Table S2: Determined oxidation degree of artificially oxidized vivianites. Comparison between photometric ferrozine method after acid digestion in 6 M HCl and STXM measurements followed by LCF of the surface (OD: 0.05 – 0.1) and bulk (OD: >0.9). The precision of the linear combination fitting approach are in the of few percent, which is important particularly for interpreting the results of the surface regions.[7]

SAMPLE (NOMINAL)	FERROZINE	STXM – BULK (OD >0.9)	STXM – SURFACE (OD 0.05-0.1)
**SynthViv (0%)**	<1%	1%	3%
**Dry (air) (10%)**	12%	8%	28%
**Air purged (20%)**	17%	21%	36%
**Anox. H_2O_2 (30%)**	24%	36%	75%
**Complete ox. (100%)**	>90%	100%	89%

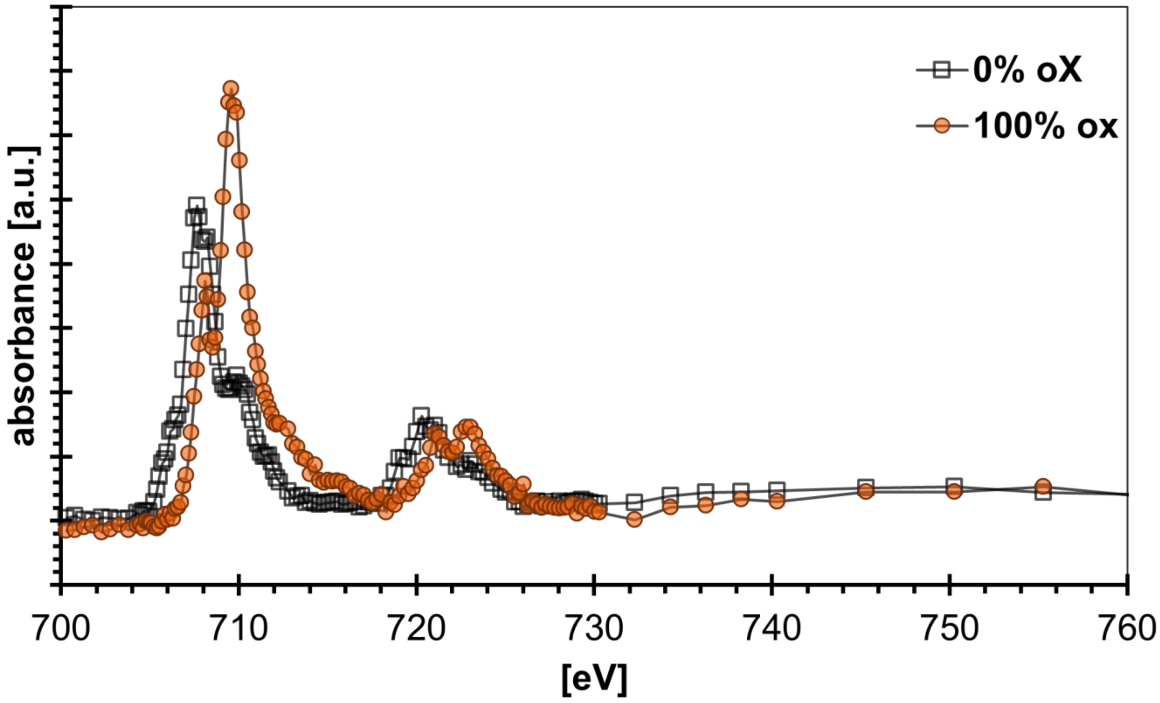

Figure S8: Two representative Fe2p edge XANES spectra of STXM measurement (averaged for OD: 0.1 – 0.9) of pristine (0% ox) and complete oxidized (100% ox) vivianite.

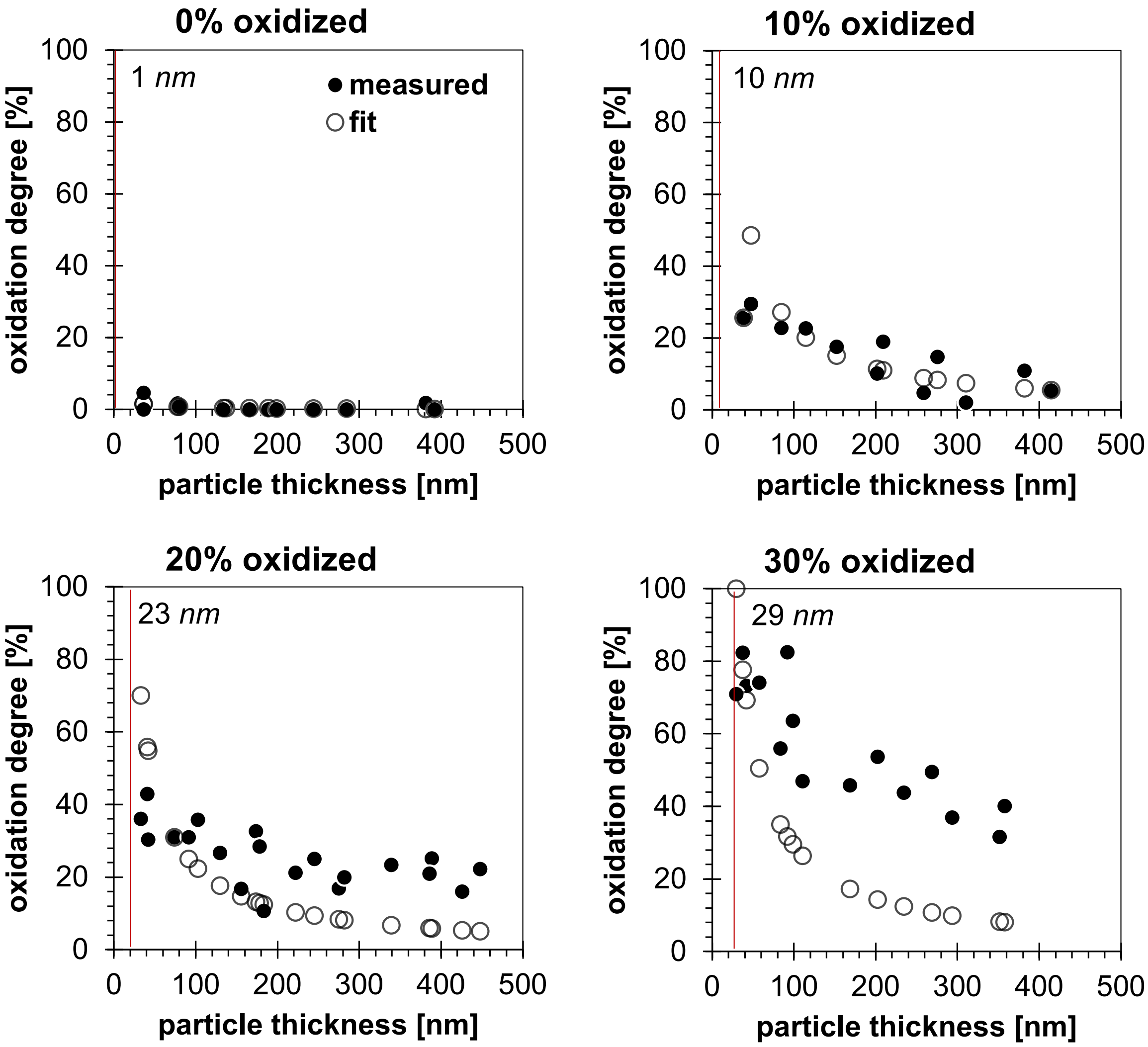

Figure S9: Oxidation degree as function of particle thickness of the 0%, 10%, 20% and 30% artificially oxidized vivianite STXM samples. Filled black circles represent the measured oxidation degree, open circles represent the fitted data according to Eq 4 (main text). In each graph, shows the red vertical line and the adjacent number the fitted thickness (m) of the 100 % oxidized shell.

3. Vivianite oxidation kinetics

3.1. Oxidation of dry vivianite under oxic conditions

The measured oxidation rate in our experiment was slower than previously reported by Rouzies and Millet [8] (Figure S10). Since i) the experimental duration in our study was short compared to Rouzies and Millet [8] (50 *vs.* 375 days), and ii) initial oxidation rates are the fastest, an over- rather than an underestimation of oxidation rates would be expected. The discrepancy between the two studies might be attributed to the different vivianite synthesis protocols. Rouzies and Millet [8] used $Fe(NH_4)_2SO_4 \cdot 6H_2O$ as Fe^{2+} source and an acetate buffered PO_4 solution. Further, even though particle size and surface area were not reported, a larger crystal size may be expected, since the synthesis solution was equilibrated

with the precipitates for 2 days, promoting crystal growth. Further, Rouzies and Millet [8] used Mössbauer spectroscopy to determine the Fe(III)/Fe(tot) ratio, which is affected by the fitting parameters applied. Furthermore, due to the small sample size of only five time points and the lack of duplicates, precision may be in doubt, especially since the present study found substantial variation even between triplicates. However, the difference between the two studies reveals the variability of vivianite oxidation rates and potentially high number of influencing factors.

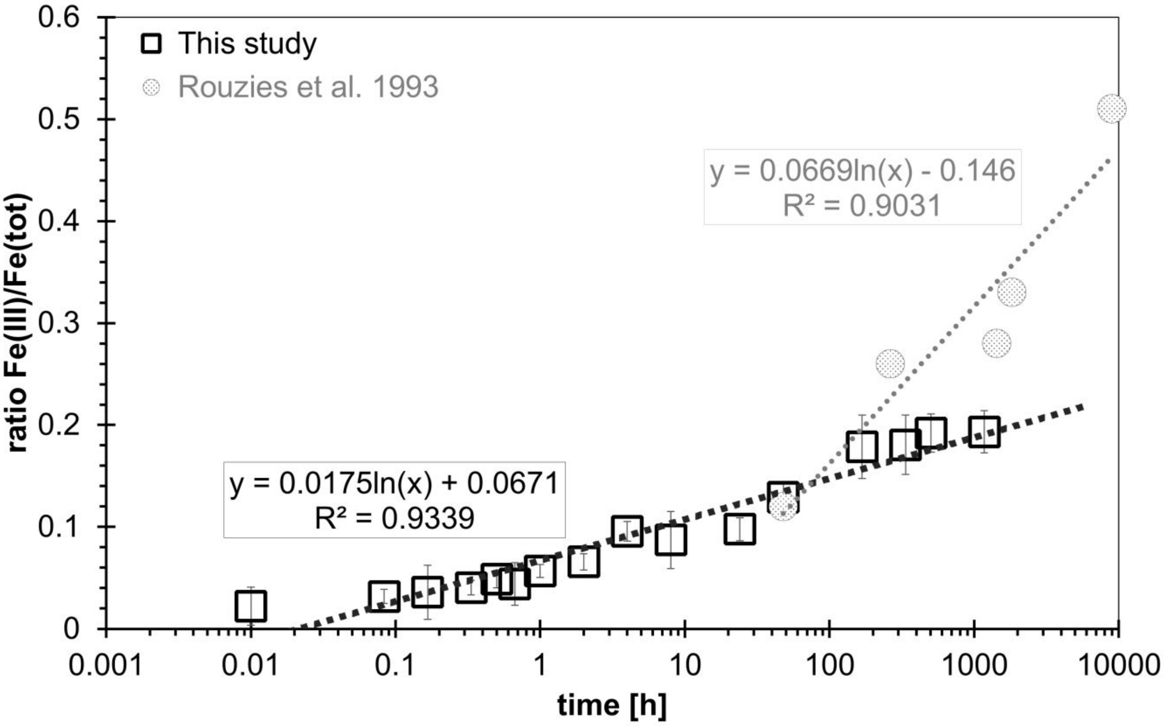

Figure S10: Oxidation degree (Fe(II)/Fe(tot)) of synthesized dry vivianite powder under atmospheric conditions (21 ± 1 °C) in the dark as a function of time. Error bars indicate standard deviation of triplicates. For comparison, results of Rouzies and Millet [8] in light grey.

3.2. Vivianite oxidation mechanism and kinetics in suspension

The oxidation kinetics of vivianite do not fit simple rate laws, but initial data (0-4 h) could be linearized by plotting the solid oxidation ratio (Fe(III)/Fe(tot)) versus the square root of time (Figure S11a), indicating diffusion controlled kinetics.[9] This is in agreement with Roldán, et al. [10], who suggested a parabolic diffusion law where the rate-limiting process is intra- or interparticle diffusion.

Assuming a shrinking-core model; here for spherical particles, three steps could be rate-controlling: i) diffusion of O_2 to the particle surface, ii) diffusion of educt or product through the solid layer (O_2, Fe(II), H_2O), and iii) the actual oxidation reaction. The three steps can be described by Eq S1, S2 and S3:[11]

$$t_X = \tau_{gas} X \tag{S1}$$

$$t_X = \tau_{diff}\left[1 - 3(1-X)^{\frac{2}{3}} + 2(1-X)\right] \tag{S2}$$

$$t_X = \tau_{ch}\left[1 - (1 - X)^{\frac{1}{3}}\right] \qquad \text{(S3)}$$

With X representing the conversion fraction; here the oxidation degree (Fe(III)/Fe(tot)), t_X the time required for reaching oxidation ratio X, and τ_{gas}, τ_{diff} and τ_{ch} the times required for complete oxidation (X=1) by gas diffusion, diffusion through the solid layer and the chemical reaction, respectively, extrapolated from measured data. The comparison of experimental oxidation data with the ideal gas diffusion (not shown), educt/product diffusion (Figure S11b) and chemical reaction (Figure S11c) models gave the best fit with the educt/product diffusion model, but model accuracy was still not satisfactory. Therefore, simple multistep kinetics in the form of a parallel reaction, were applied, according to Eq S4: [12]

$$\frac{X_t}{X_\infty} = w_1(1 - e^{-a_1 t^{n_1}}) + w_2(1 - e^{-a_2 t^{n_2}}) \qquad \text{(S4)}$$

with X_t: oxidation degree at time t; X_∞: oxidation degree at equilibrium; w_1 and w_2: weighting factor for the importance of each reaction ($w_1 + w_2 = 1$); a_1, a_2: fitting parameters and n_1, n_2: kinetic exponents/shape factor.[13] The experimental data could be fitted very well, but it includes a large number of fitting parameters (Figure S11d). The values of n_1 and n_2 define the type of reaction mechanism; reactions with i) $n < 1$ are considered diffusion-controlled, ii) $1 \approx n < 2$ are considered controlled by first-order kinetics and iii) $n \approx 2$ are considered nucleation-controlled.[12] Fitting resulted for all temperatures except 75 °C in n values < 1 (n_1: 0.33 ± 0.08; n_2: 0.33 ± 0.09) with n increasing with increasing temperature, again suggesting a diffusion-controlled mechanism. Only at 75 °C n_1 equaled 1.12, suggesting first-order kinetic control. Therefore, a diffusion controlled kinetic model seems to describe the central mechanism for vivianite oxidation best.

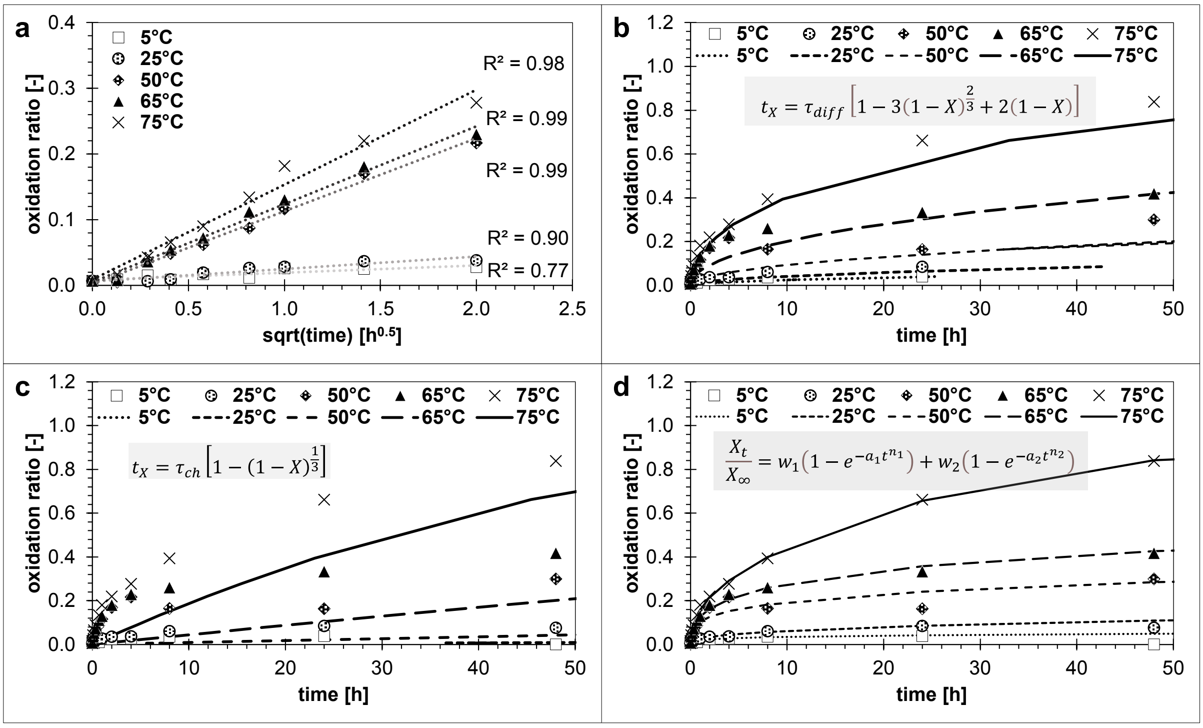

Figure S11: Comparison of experimental vivianite oxidation data at various temperatures (5 – 75 °C) as a function of time (symbols) with fits using different models (lines); equations are given in each plot. a) parabolic model, linearization of data using square-root of time (0-4 h); b) shrinking-core model (diffusion controlled, through a solid layer); c) shrinking-core model (chemical reaction controlled); d) Parallel reaction model. Experimental conditions: 200 µM vivianite at pH 6.0 (10 mM MES, IS=10 mM) under oxic conditions.

In diffusion-controlled reactions, the rate of product formation decreases proportionally with the thickness of the product barrier layer.[9] The diffusion controlled reaction kinetics can be described by several models, considering different shape factors, represented in Figure S12 and Table S3: a) infinite flat plane (1D), b) cylindrical (diffusion occurs radially through a cylindrical shell, 2D), c) spherical (paraboloid law, 3D) and d) spherical (radial diffusion in a sphere, 3D). A summary and derivation of each model can be found in Khawam and Flanagan [9].

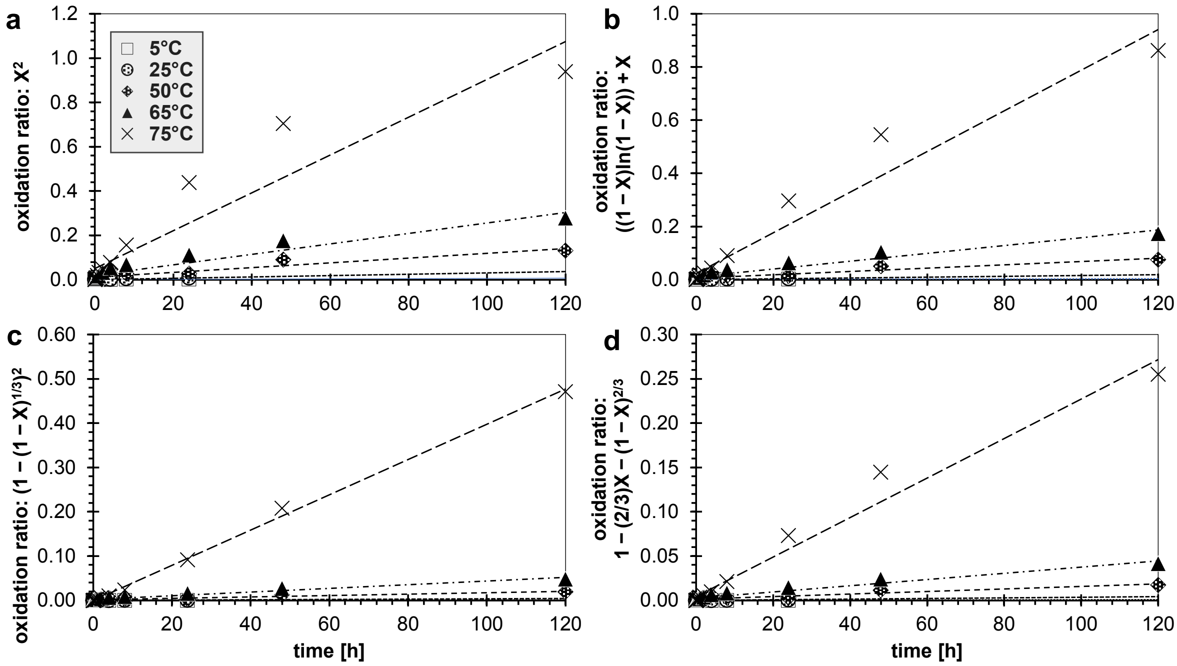

Figure S12: Linearization of experimental vivianite oxidation data at various temperatures (5 – 75 °C) according to different diffusion models as a function of time. Dotted lines represent the linear regression lines for each temperature. Respective functions and R^2 values can be found in Table S3.

Table S3: Diffusion models used in Figure S10 a-d with function of linear regression line and R^2 value.

**Diffusion models**: $Differential\ form: f(x) = \frac{1dx}{kdt}$; $integral\ form: g(x) = kt$		
**a) 1D diffusion**	$f(x) = \frac{1}{2x}$	$g(x) = x^2$
temperature	linear regression	R²
5°C	y = 0.00006x + 0.00031	0.72
25°C	y = 0.00031x + 0.00034	0.97
50°C	y = 0.0011x + 0.012	0.86
65°C	y = 0.0024x + 0.020	0.92
75°C	y = 0.0085x + 0.050	0.89
**b) 2D diffusion**	$f(x) = -\left[\frac{1}{\ln(1-x)}\right]$	$g(x) = ((1-x)\ln(1-x)) + x$
temperature	linear regression	R²
5°C	y = 0.00003x + 0.00015	0.73
25°C	y = 0.00016x + 0.00017	0.97
50°C	y = 0.00062x + 0.0063	0.87
65°C	y = 0.0015x + 0.011	0.94
75°C	y = 0.0077x + 0.023	0.95
**c) 3D diffusion−Jander**	$f(x) = \frac{\left[3(1-x)^{\frac{2}{3}}\right]}{\left[2\left(1-(1-x)^{\frac{1}{3}}\right)\right]}$	$g(x) = \left(1-(1-x)^{\frac{1}{3}}\right)^2$
temperature	linear regression	R²
5°C	y = 0.000007x + 0.00004	0.73

25°C	y = 0.000036x + 0.00004	0.97
50°C	y = 0.00016x + 0.0014	0.89
65°C	y = 0.00041x + 0.0024	0.96
75°C	y = 0.0040x - 0.00069	1.00
**d) Ginstling− Brounshtein**	$f(x) = \frac{3}{\left[2\left((1-x)^{-\frac{1}{3}} - 1\right)\right]}$	$g(x) = 1 - \left(\frac{2}{3}\right)x - (1-x)^{\frac{2}{3}}$
temperature	linear regression	R^2
5°C	y = 0.000007x + 0.00003	0.73
25°C	y = 0.000036x + 0.00004	0.97
50°C	y = 0.00014x + 0.0014	0.88
65°C	y = 0.00035x + 0.0024	0.95
75°C	y = 0.0022x + 0.0042	0.98

To determine a diffusion coefficient, Sidhu, et al. [14] developed a kinetic model for the oxidation of the mixed valence Fe mineral magnetite ($Fe^{2+}(Fe^{3+})_2O_4$), which has been successfully applied in several studies [12,14-16] *a.o.* the aqueous oxidation at low temperatures.[15] The model is based on the radial diffusion in a sphere with a constant diffusion coefficient (D) in [m^2 h^{-1}], under non-steady state conditions (moving boundary) Eq S5: [17]

$$\frac{\partial C}{\partial t} = D\left(\frac{\partial^2 C}{\partial r^2} + \frac{\frac{2}{r}\partial C}{\partial r}\right) \tag{S5}$$

where C is the reactant concentration [mol L^{-1}], and r is the radius [m]. Importantly, Sidhu, et al. [14] considers for magnetite oxidation the outward diffusion of Fe(II) to the surface; hence C = Fe(II) concentration. Due to structural differences and the coordination of FeO_6 octahedra with PO_4 tetrahedra, for vivianite, O_2 diffusion inwards is more likely. Further, it is assumed that Fe(II) oxidizes immediately at the surface and C = 0. The O_2 concentration is in excess and does not limit the reaction rate (constant surface concentration), and hence, does not appear in this model.[16,17]

Considering these boundary conditions, integration and rearrangement results for small times in Eq S6. For derivation and further explanations, it is referred to Crank [17] and Sidhu, et al. [14]

$$\frac{X_t}{X_\infty} \cdot \frac{1}{t} = 6\pi^{-\frac{1}{2}}\left(\frac{D}{r^2}\right)^{\frac{1}{2}} \cdot \frac{1}{t^{\frac{1}{2}}} - 3\frac{D}{r^2} \tag{S6}$$

With X_t: oxidation degree at time t; X_∞: oxidation degree at equilibrium (complete oxidation = 1). Hence, plotting $\frac{X_t}{X_\infty} \cdot \frac{1}{t}$ vs $\frac{1}{t^{\frac{1}{2}}}$ should give a straight line, which corresponds well with our data (Figure S13). If r is known ($\frac{9.5\ \mu m}{2}$; according to the particle size measurement of synthesized vivianite[3]), the diffusion

coefficient D can be calculated from the slope $\left(6\pi^{-\frac{1}{2}}\left(\frac{D}{r^2}\right)^{\frac{1}{2}}\right)$ and the intercept $\left(-3\frac{D}{r^2}\right)$, both calculated values are given in Figure S13. To account for the reaction stoichiometry of O_2 with Fe(II), the D values were divided by 4, and by a factor accounting for the O_2 saturation in water at different temperatures. The effect of temperature on diffusion coefficient D can be described by an Arrhenius-type equation[14] (Eq S7):

$$D = D_0 \cdot e^{-\frac{E_a}{RT}} \qquad \text{(S7)}$$

where D_0 is the frequency factor [$m^2\ h^{-1}$], E_a is the activation energy [$J\ mol^{-1}$], R is the gas constant [$J\ K^{-1}\ mol^{-1}$] and T is the temperature in [K]. The average of the D^a (slope) and D^b (intercept) (Table in Figure S13) was used, and by plotting $ln(D)$ as a function of T^{-1}, an Arrhenius plot was obtained (Figure S14), from which $E_a = 34.2 \pm 8.1$ kJ mol^{-1} and the frequency factor (D_0) $3.8 \pm 2.9 \cdot 10^{-8}$ $cm^2\ sec^{-1}$ were determined (Eq S7). The E_a for diffusion is lower than the value which was determined for the overall reaction 77.6 ± 10.3 kJ mol^{-1}, and also lower than the reported E_a for the oxidation of magnetite, ranging between 79.5 and 100 kJ mol^{-1},[12,14,15,18] and a D_0 of 3.16 x 10^{-5} $cm^2\ sec^{-1}$.[14] The comparatively low E_a of vivianite may be related to the easier diffusion of O_2 into the crystal structure, which is facilitated by the layered structure of vivianite,[19] compared to the outward diffusion of Fe for magnetite.[14]

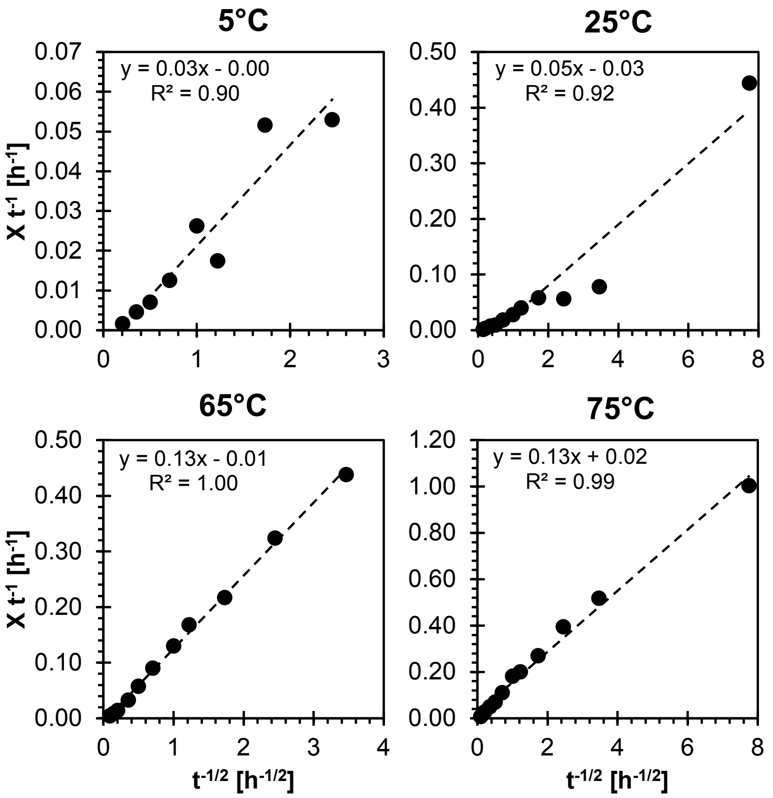

Temp. [°C]	D^a [$cm^2\ s^{-1}$]	D^b [$cm^2\ s^{-1}$]
5	2.1 10^{-14}	8.8 10^{-14}
25	9.8 10^{-14}	6.1 10^{-13}
50	4.8 10^{-13}	2.5 10^{-13}
65	5.7 10^{-13}	1.4 10^{-13}
75	5.7 10^{-13}	4.6 10^{-13}

Figure S13: Linearization of temperature dependent oxidation of vivianite. Oxidation ratio per time [h^{-1}] versus the reciprocal square root of time [$h^{-0.5}$] according to a spherical diffusion model.[14,17] The dotted line indicates

the linear regression line. The table shows the calculated values for the diffusion coefficient (D), calculated by the slope (D^a) or the y-axis intersection (D^b). To account for O_2 diffusion, a correction for the temperature-dependent dissolved O_2 concentration was included and diffusion coefficients were divided by 4, according to the stoichiometry of Fe oxidation by O_2.

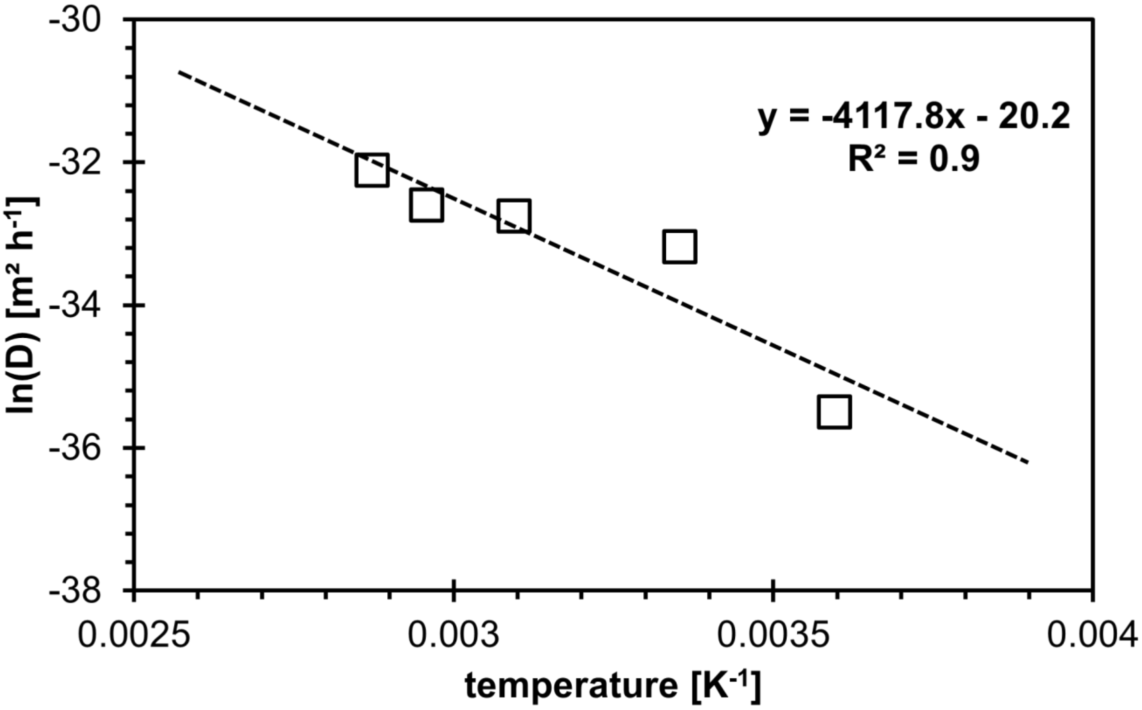

Figure S14: Oxidation kinetics of suspended vivianite (200 µM) over an ambient temperature range (5-75 °C) under atmospheric conditions at pH 6.0 (10 mM MES, IS=10 mM). Arrhenius-type plot for vivianite oxidation; natural logarithm of the determined diffusion coefficients (m^{-2} h^{-1}) as a function of the reciprocal temperature (5-75 °C) in Kelvin (K). Activation energy: E_a = 34.2 ± 8.1 kJ mol^{-1}.

4. Dissolution of pre-oxidized vivianite under anoxic conditions

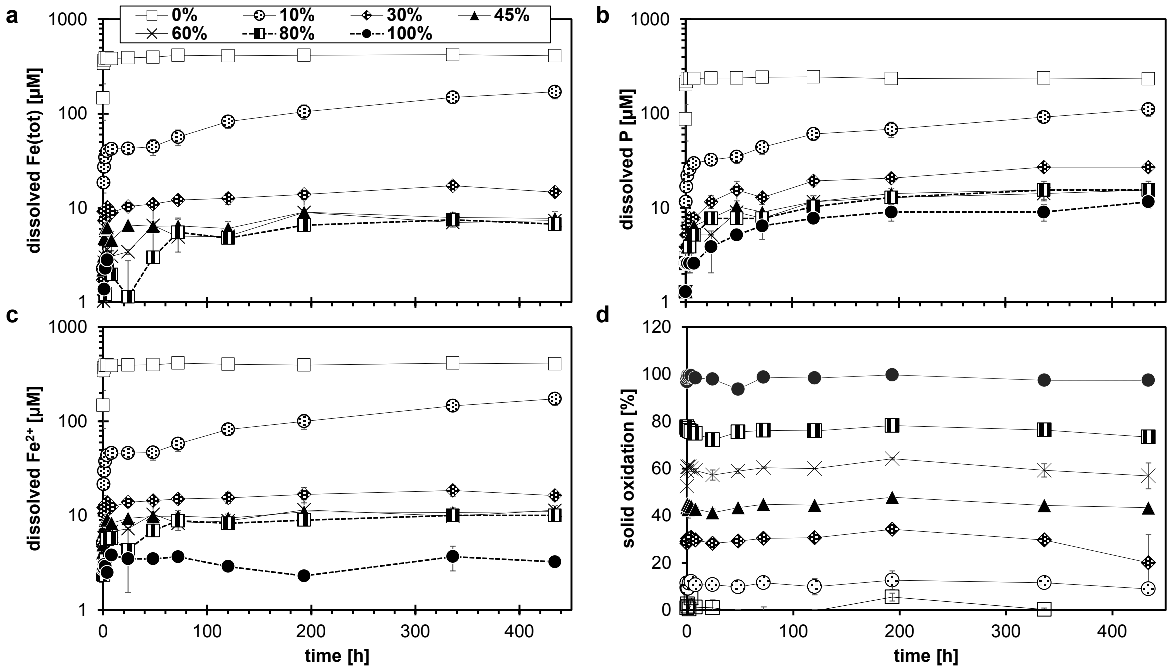

Figure S15: Dissolution of vivianite (1 mM) under anoxic conditions in buffered solution (IS=10 mM) at pH 6.0 which was previously oxidized to a certain degree (0-100% of Fe(tot)) with diluted H_2O_2 solution. a) dissolved Fe(tot), b) dissolved P, c) dissolved Fe^{2+}, d) solid oxidation degree [%]. Error bars indicate deviation between duplicates.

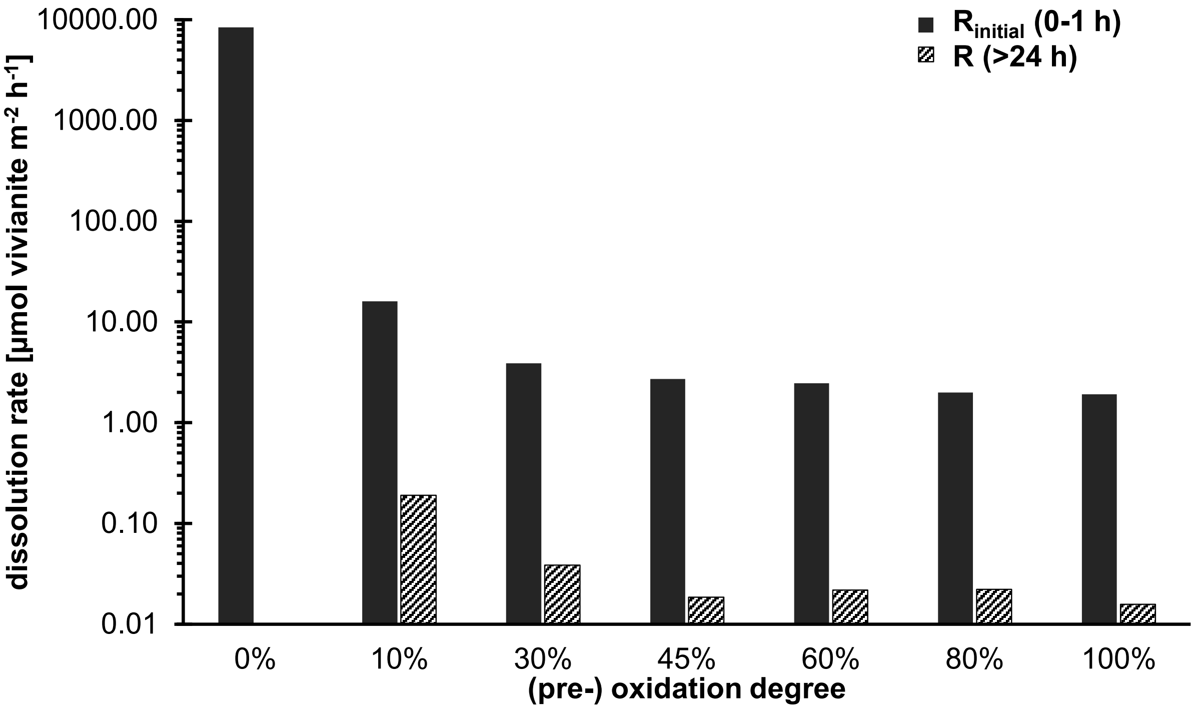

Figure S16: Dissolution rate (R) and initial dissolution rate ($R_{initial}$) of vivianite, where stock suspensions had been oxidized to a certain degree with diluted H_2O_2. The dissolution experiment was conducted under anoxic

conditions (N_2 atmosphere) at pH 6 (10 mM MES; ionic strength = 10 mM adjusted with NaCl) with 200 µM vivianite suspensions.

5. Vivianite dissolution under oxic conditions

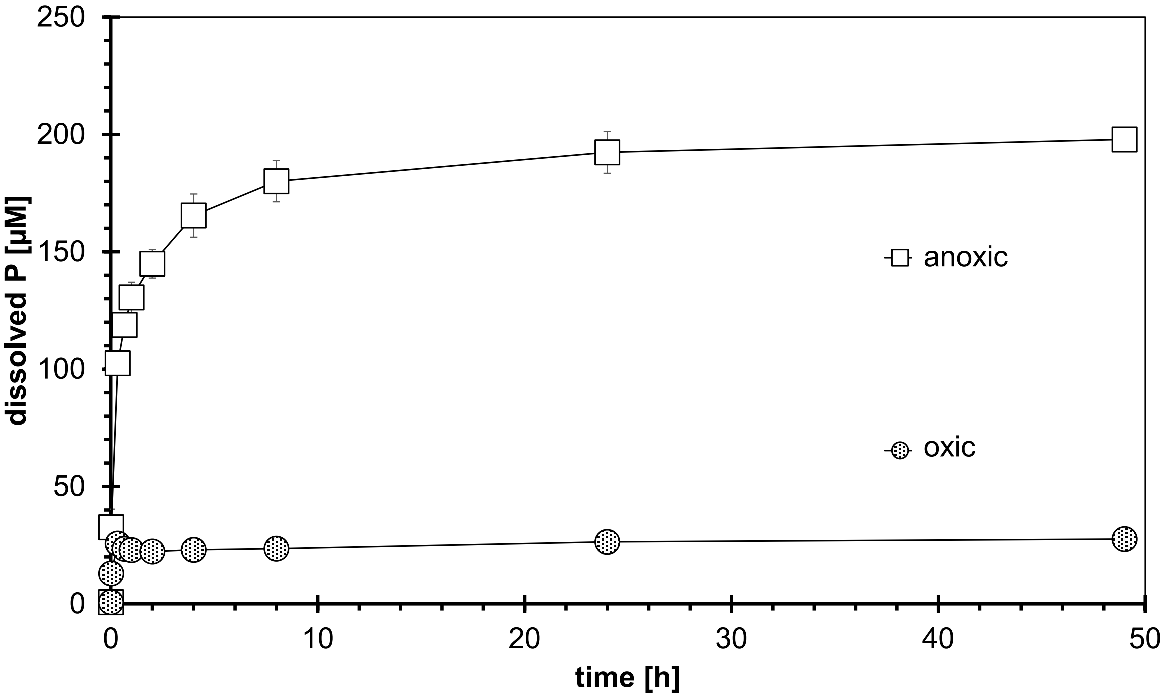

Figure S17: Comparison of initially pristine vivianite dissolution under anoxic (open squares) and oxic (filled circles) conditions at pH6.0 (10 mM MES; IS=10 at room temperature 21 °C). Suspensions contained 200 µM vivianite. Error bars indicate deviations between duplicates.

Table S4: Dissolution rates of 200 µM vivianite under anoxic and oxic conditions at pH 6.0 (10 mM MES; IS=10 at room temperature 21 °C).

**vivianite dissolution (pH 6.0; IS = 10 mM)**			
	**anoxic**	**oxic**	
initial dissolution rate	7728	2971	[µmol Viv $m^{-2}h^{-1}$]
dissolution rate (8-49h)	-	0.4	[µmol Viv $m^{-2}h^{-1}$]

5.1. Flow-through dissolution experiment under oxic conditions

Continuous flow stirred tank reactors (CFSTRs) with a volume of 90 mL were used to examine the dissolution rate of vivianite (Figure S18; a detailed description of the reactor design has been previously given in Frazier, et al. [20]). CFSTRs were wrapped in aluminum foil to prevent photo-chemical reactions. The reactors were sealed with 0.1 µm membrane filter (Whatman, NC10) to retain the solids inside the reactor. To each reactor, 0.1 g of dried vivianite was added, resulting in a solid-to-solution ratio of

1.11 g L^{-1}. Oxic influent solution was pumped into the reactor using a peristaltic pump at rates that allowed a hydraulic residence time of 4 h. The influent solution had a pH of 6.0 (10 mM MES), 10 mM NaCl as electrolyte and the dissolved Fe and P concentrations were below limit of quantification (~ 1 µM). Effluent samples (1 mL) were collected at various time intervals and immediately stabilized with 1 M HCl and analyzed for dissolved Fe, Fe(II) and P concentration. Remaining effluent solution was further used to monitor the pH and the flowrate (gravimetrically). At the end of the experiment, outflow filters with remaining solids were collected and stored in the anoxic chamber for solid phase analysis.

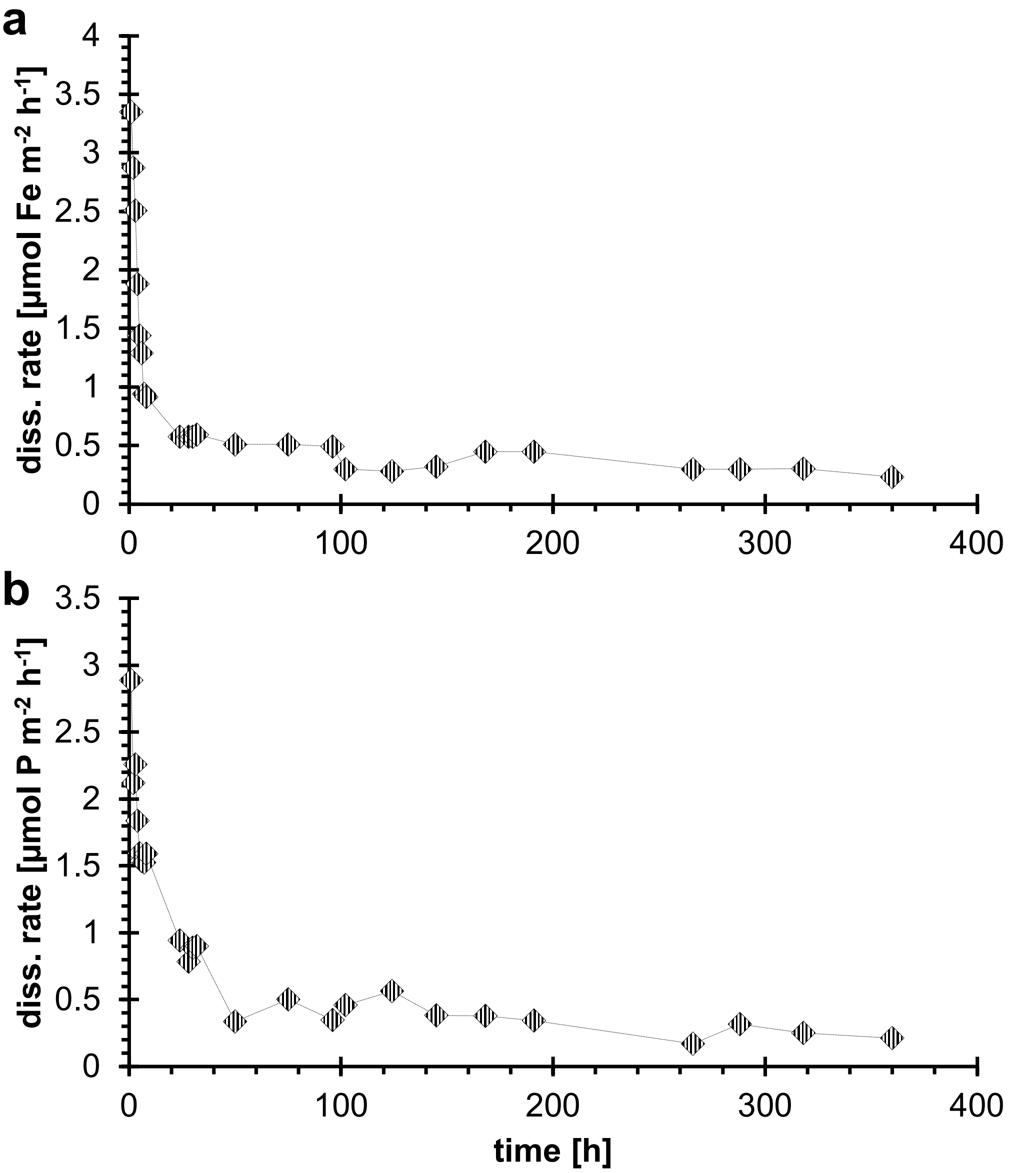

Figure S18: Continuous flow stirred tank reactors (CFSTR) dissolution experiment of 0.1 g vivianite (solid-to-solution ratio of 1.11 g L^{-1}) under oxic conditions. The Fe- and P- free 10 mM MES buffered inflow solution (pH 6.0, 10 mM NaCl). The pumping rate was kept constant at 0.33 mL min-1 (residence time ~4 h). Vivianite dissolution rate calculated from dissolved a) Fe and b) P outflow concentration considering the minerals

stoichiometry. The steady-state dissolution rate (0.2 ± 0.06 µM vivianite m⁻² h⁻¹) was calculated from the time interval 50-360 h.

5.2. The temperature dependence of vivianite dissolution under oxic conditions

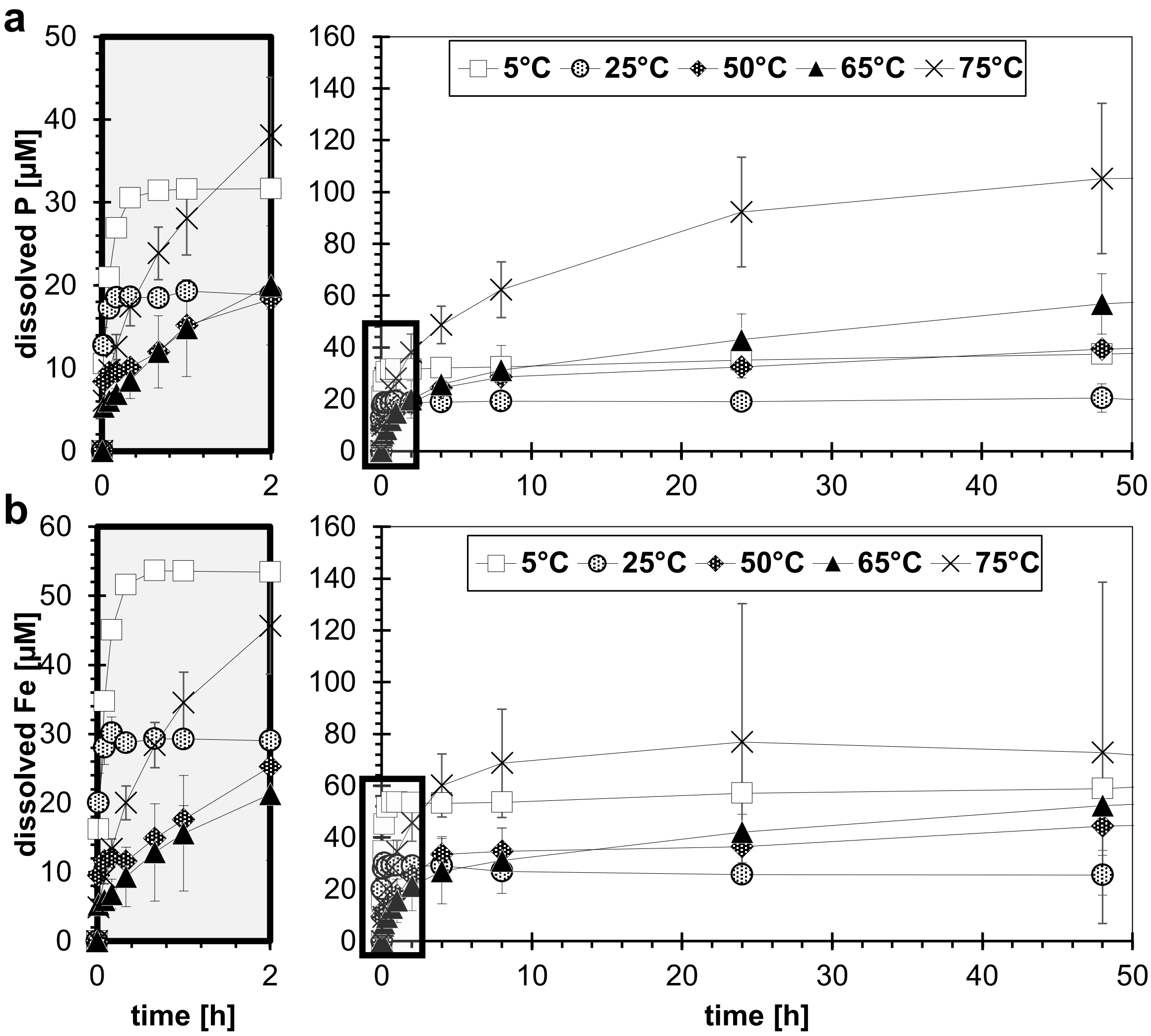

Figure S19: Dissolved a) P and b) Fe concentration as a function of time resulting from vivianite dissolution (200 µM) at various temperatures (5-75°C) under atmospheric conditions at pH 6 (10 mM MES; IS=10 mM). Error bars represent deviations between duplicates and black frame indicates magnified area, displayed in the left outtake.

5.2.1. SEM images and EDX analysis vivianite particles after dissolution under oxic conditions at different temperatures

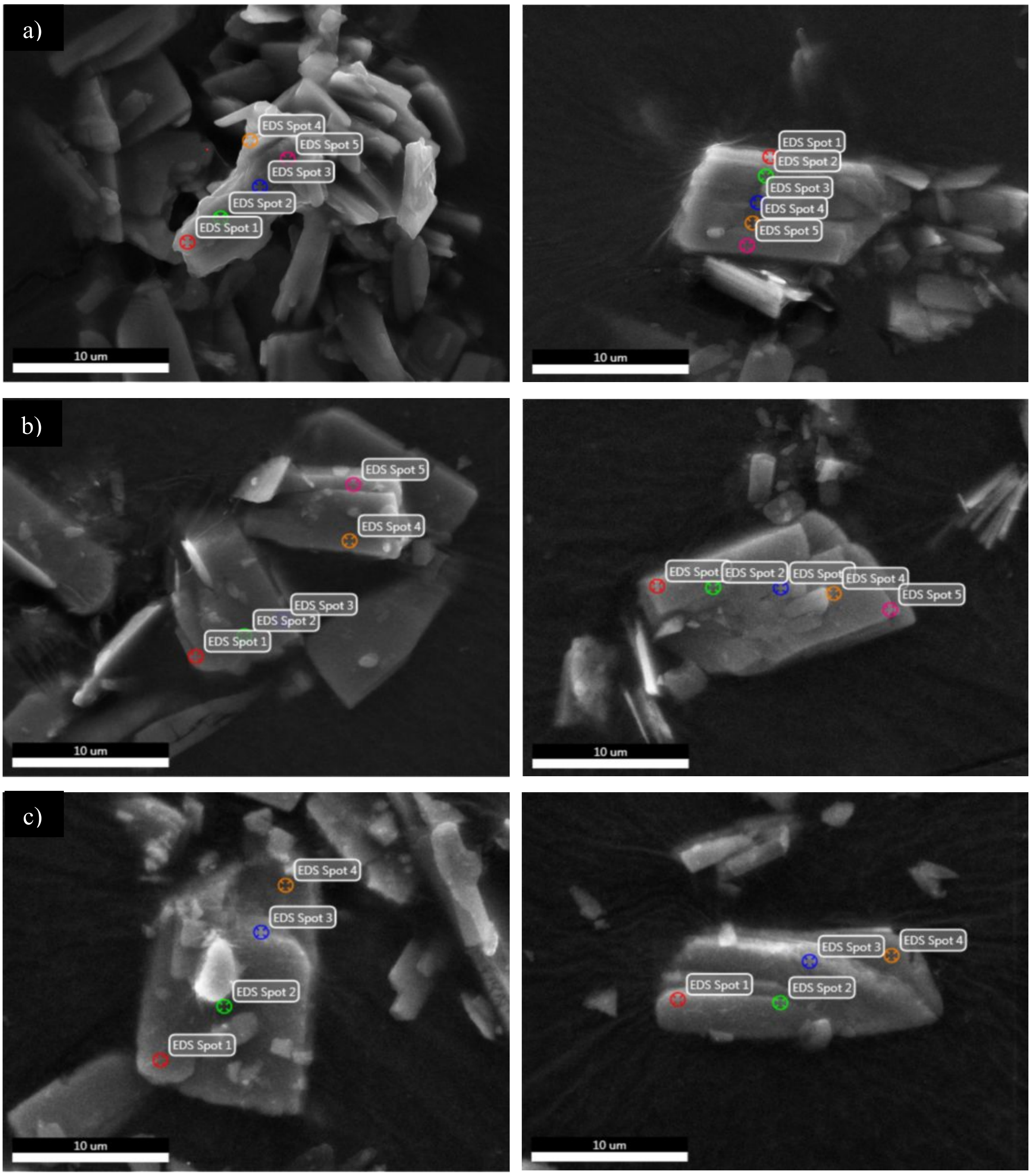

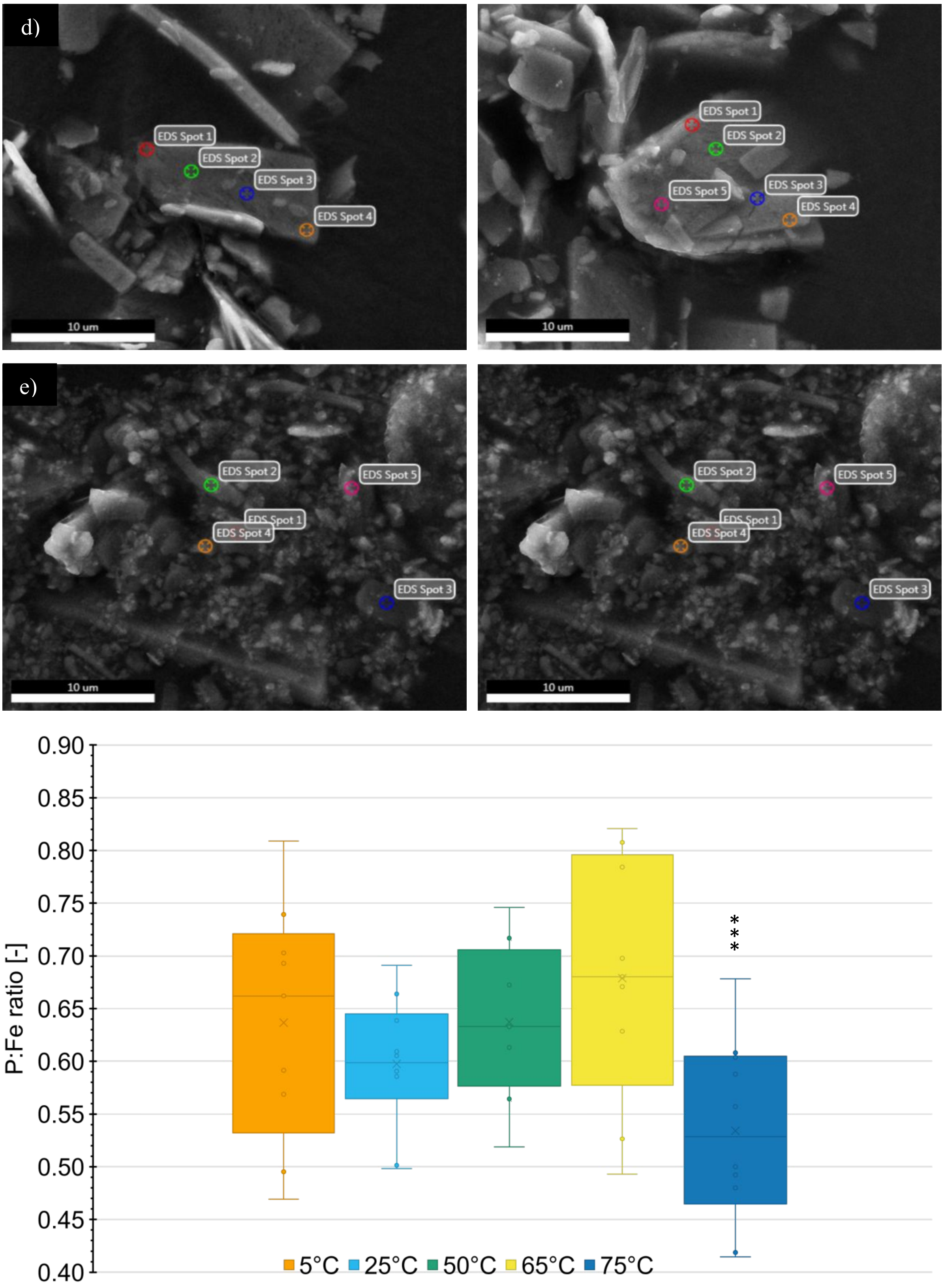

Figure S20: SEM-EDX images of oxidized vivianites at different temperature; a) 5°C, b) 25°C, c) 50°C, d) 65°C, e) 75°C. Box and whisker plot shows P:Fe ratios determined by EDX for the spots, indicated in the SEM images. Stars above the boxplot indicate significant differences (α < 0.05) between the samples.

5.3. pH dependence of vivianite dissolution under oxic conditions

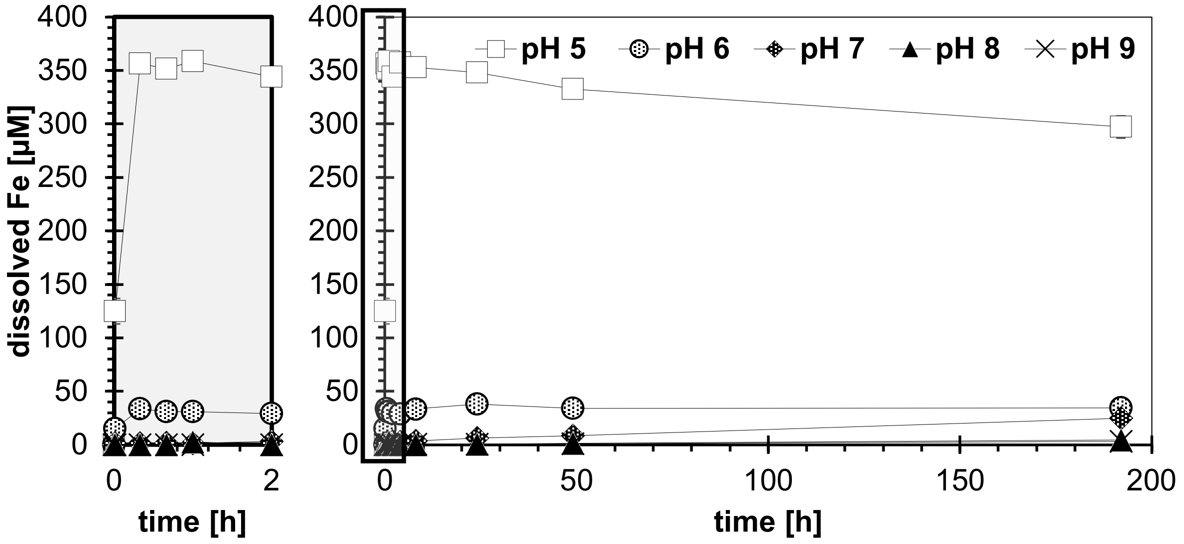

Figure S21: Dissolved Fe concentrations as a function of time upon vivianite dissolution (200 µM) over an environmentally relevant pH range (5-9) under atmospheric (oxic) conditions in buffered solutions (IS=10 mM). Error bars indicate deviations between duplicates.

6. References

1 Momma, K. & Izumi, F. VESTA 3 for three-dimensional visualization of crystal, volumetric and morphology data. *Journal of Applied Crystallography* **44**, 1272-1276 (2011). https://doi.org/10.1107/S0021889811038970

2 Capitelli, F., Chita, G., Ghiara Maria, R. & Rossi, M. in *Zeitschrift für Kristallographie Crystalline Materials* Vol. 227 92 (2012). http://doi.org/10.1524/zkri.2012.1442

3 Metz, R., Kumar, N., Schenkeveld, W. D. C. & Kraemer, S. M. Rates and Mechanism of Vivianite Dissolution under Anoxic Conditions. *Environmental Science & Technology* (2023). https://doi.org/10.1021/acs.est.3c04474

4 Kubeneck, L. J., ThomasArrigo, L. K., Rothwell, K. A., Kaegi, R. & Kretzschmar, R. Competitive incorporation of Mn and Mg in vivianite at varying salinity and effects on crystal structure and morphology. *Geochimica et Cosmochimica Acta* **346**, 231-244 (2023). https://doi.org/10.1016/j.gca.2023.01.029

5 Dormann, J.-L., Gasperin, M. & Poullen, J.-F. Étude structurale de la séquence d'oxydation de la vivianite Fe3 (PO4) 2• 8 H2O. *Bulletin de Minéralogie* **105**, 147-160 (1982). https://doi.org/10.3406/bulmi.1982.7597

6 Voegelin, A., Kaegi, R., Frommer, J., Vantelon, D. & Hug, S. J. Effect of phosphate, silicate, and Ca on Fe(III)-precipitates formed in aerated Fe(II)- and As(III)-containing water studied by X-ray absorption spectroscopy. *Geochimica et Cosmochimica Acta* **74**, 164-186 (2010). https://doi.org/10.1016/j.gca.2009.09.020

7 Dynes, J. J., Tyliszczak, T., Araki, T., Lawrence, J. R., Swerhone, G. D. W., Leppard, G. G. & Hitchcock, A. P. Speciation and Quantitative Mapping of Metal Species in Microbial Biofilms Using Scanning Transmission X-ray Microscopy. *Environmental Science & Technology* **40**, 1556-1565 (2006). 10.1021/es0513638

8 Rouzies, D. & Millet, J. M. M. Mössbauer study of synthetic oxidized vivianite at room temperature. *Hyperfine Interactions* **77**, 19-28 (1993). https://doi.org/10.1007/BF02320295

9 Khawam, A. & Flanagan, D. R. Solid-State Kinetic Models: Basics and Mathematical Fundamentals. *The Journal of Physical Chemistry B* **110**, 17315-17328 (2006). 10.1021/jp062746a

10 Roldán, R., Barrón, V. & Torrent, J. Experimental alteration of vivianite to lepidocrocite in a calcareous medium. *Clay Minerals* **37**, 709 (2002). http://doi.org/10.1180/0009855023740072

11 Levenspiel, O. *Chemical reaction engineering*. (John Wiley & Sons, 1998).

12 Monazam, E. R., Breault, R. W. & Siriwardane, R. Kinetics of Magnetite (Fe_3O_4) Oxidation to Hematite (Fe_2O_3) in Air for Chemical Looping Combustion. *Industrial & Engineering Chemistry Research* **53**, 13320-13328 (2014). http://doi.org/10.1021/ie501536s

13 Monazam, E. R., Breault, R. W., Siriwardane, R., Richards, G. & Carpenter, S. Kinetics of the reduction of hematite (Fe2O3) by methane (CH4) during chemical looping combustion: A global mechanism. *Chemical Engineering Journal* **232**, 478-487 (2013). https://doi.org/10.1016/j.cej.2013.07.091

14 Sidhu, P. S., Gilkes, R. J. & Posner, A. M. Mechanism of the low temperature oxidation of synthetic magnetites. *Journal of Inorganic and Nuclear Chemistry* **39**, 1953-1958 (1977). https://doi.org/10.1016/0022-1902(77)80523-X

15 Tang, J., Myers, M., Bosnick, K. A. & Brus, L. E. Magnetite Fe_3O_4 Nanocrystals: Spectroscopic Observation of Aqueous Oxidation Kinetics. *The Journal of Physical Chemistry B* **107**, 7501-7506 (2003). http://doi.org/10.1021/jp027048e

16 Li, Z., Chanéac, C., Berger, G., Delaunay, S., Graff, A. & Lefèvre, G. Mechanism and kinetics of magnetite oxidation under hydrothermal conditions. *RSC Advances* **9**, 33633-33642 (2019). 10.1039/C9RA03234G

17 Crank, J. *The Mathematics of Diffusion*. 2 edn, 89-104 (Clarendon Press, 1979).

18 Monsen, B. E., Olsen, S. E. & Kolbeinsen, L. Kinetics of magnetite oxidation. *Scandinavian journal of metallurgy* **23**, 74-80 (1994).

19 Hanzel, D., Meisel, W., Hanzel, D. & Gütlich, P. Mössbauer effect study of the oxidation of vivianite. *Solid State Communications* **76**, 307–310 (1990). https://doi.org/10.1016/0038-1098(90)90843-Z

20 Frazier, S. W., Kretzschmar, R. & Kraemer, S. M. Bacterial Siderophores Promote Dissolution of UO2 under Reducing Conditions. *Environmental Science & Technology* **39**, 5709-5715 (2005). https://doi.org/10.1021/es050270n
